# Green-Synthesized Silver Nanoparticles Mediated by the Marine Macroalgae *Chaetomorpha linum*: Physicochemical Characterization, Antimicrobial and Antioxidant Activities

**DOI:** 10.3390/ma19194119

**Published:** 2026-09-26

**Authors:** Hatice Banu Keskinkaya

**Affiliations:** 1 Department of Biotechnology, Faculty of Science, Necmettin Erbakan University, Konya 42090, Türkiye; dr.haticebanu@gmail.com; Tel.: +90-507-711-88-90; 2 Application and Research Center for Medicinal and Cosmetic Plants, Necmettin Erbakan University, Konya 42090, Türkiye

**Keywords:** biomaterials, biological activity, *Chaetomorpha linum*, green synthesis, marine macroalgae, silver nanoparticles

## Abstract

The green synthesis of silver nanoparticles (AgNPs) using marine macroalgae represents an environmentally friendly alternative to conventional chemical methods. In this study, silver nanoparticles (CL-AgNPs) were successfully synthesized using the aqueous extract of the marine green macroalga *Chaetomorpha linum*. The extract was characterized by HPLC-DAD and FTIR, revealing phenolic compounds and functional groups involved in nanoparticle reduction and stabilization. The synthesized CL-AgNPs were characterized by UV–Vis, FTIR, XRD, FESEM, EDS and DLS analyses, confirming the formation of predominantly spherical nanoparticles with an average particle size of 31.09 ± 2.52 nm (range: 21.26–42.90 nm) and a face-centered cubic crystalline structure. The antimicrobial and antioxidant activities of the synthesized nanoparticles were comprehensively evaluated and directly compared with those of the corresponding crude algal extract. Compared with the crude extract, CL-AgNPs exhibited enhanced antimicrobial activity, with minimum inhibitory concentration (MIC) values as low as 1.56 μg/mL, together with improved antioxidant activity in DPPH, ABTS, CUPRAC, and metal chelating assays. These findings demonstrate that *C. linum*-mediated AgNPs constitute a sustainable and promising nanoplatform for biomedical, pharmaceutical, food, and environmental applications.

## 1. Introduction

Macroalgae have gained considerable scientific and commercial attention in recent years due to their rich nutritional composition and broad-spectrum antimicrobial potential. These marine biomass sources are characterized by substantial levels of polysaccharides, fatty acids, vitamins, pigments (including carotenoids, phycobiliproteins and chlorophyll derivatives) and diverse phenolic constituents, which collectively confer high biological value and multifunctional bioactivity. Driven by these attributes, macroalgae have been increasingly exploited as natural platforms for food, pharmaceutical, cosmetic and biotechnological applications. The growing interest has further extended to nanotechnology-based innovations, wherein algal metabolites serve as reducing, stabilizing or capping agents in the synthesis of nanoscale materials (<100 nm or assembled nanostructures). Consequently, algae-assisted nanomaterial production has emerged as an attractive, sustainable and eco-compatible strategy, enabling the development of high-performance nanostructures with antimicrobial, antioxidant and functional biomaterial properties [1]. Macroalgae represent an emerging reservoir of functional biomolecules and have become a key focus in sustainable biotechnology due to their nutritional richness and diverse bioactivities. In addition to high levels of polysaccharides, fatty acids, pigments and phenolic compounds, macroalgae exhibit antimicrobial, antioxidant and anti-inflammatory properties, which collectively underpin their expanding commercial relevance across food, pharmaceutical, cosmeceutical and biomaterial sectors. Recent advances have extended this interest toward nano-enabled applications, particularly in the biosynthesis of high-value metallic nanostructures.

In green nanotechnology, various algal formats—ranging from crude extracts to live biomass cultures—can serve as highly effective reducing, stabilizing or capping agents in the fabrication of metal nanoparticles. The biosynthesis of silver nanoparticles (AgNPs) using algal systems is known to be influenced by several physicochemical and environmental parameters, including extract concentration, precursor levels, pH, incubation time, illumination and temperature [2]. Among the diverse macroalgal taxa, the green seaweed *Chaetomorpha linum* (Ulvophyceae, Cladoporales, Cladoporaceae), which is widely distributed along the Turkish coastal regions, particularly in the Aegean and Mediterranean Seas, has attracted interest due to its biochemical versatility and ecological abundance. In light of its potential in nanotechnological applications, clarifying the phylogenetic position and systematic taxonomy of *C. linum* is essential; the relevant classification is summarized in Table 1.

Nanotechnology encompasses the manipulation, design and controlled assembly of materials at the 1–100 nm scale and has propelled the development of novel nanosystems for biomedical, environmental and industrial applications [4]. Among metal-based nanomaterials, silver nanoparticles hold a prominent position due to their unique physicochemical and biological properties, which enhance their applicability compared to other metallic nanostructures [1,5]. To date, AgNPs have been synthesized via physical, chemical and biological routes; however, conventional approaches often rely on high energy inputs and toxic reagents, limiting scalability and raising environmental concerns. In contrast, biological or “green” synthesis strategies employing bacteria, algae, fungi or plants offer low-cost, eco-compatible and efficient alternatives with minimal hazardous waste [2,6]. These advantages align with the expanding demand for sustainable materials and have renewed interest in algal-mediated AgNP production.

Moreover, silver has been historically recognized for its antimicrobial efficacy, with silver-based compounds being utilized in infection control since the 19th century. Contemporary nanosilver platforms further enhance antimicrobial potency, broadening their applicability to medical devices, wound dressings, coatings and water treatment technologies. Despite these advancements, research gaps remain in identifying suitable algal species and optimizing synthesis parameters to yield biofunctional, application-ready nanostructures.

Numerous studies have demonstrated the feasibility of synthesizing silver nanoparticles using marine macroalgae as renewable biological resources. Green AgNPs have been successfully fabricated using species such as *Sargassum muticum*, *Ulva lactuca*, *Gracilaria corticata*, *Turbinaria conoides*, *Chaetomorpha antennina*, and *Chaetomorpha ligustica*, yielding nanoparticles with promising antimicrobial, antioxidant, anticancer, and catalytic properties [7,8,9,10,11]. These studies demonstrate the versatility of marine macroalgae as reducing and stabilizing agents for nanoparticle synthesis while highlighting the growing interest in environmentally sustainable nanotechnology.

Despite these encouraging developments, significant knowledge gaps remain regarding the relationship between the phytochemical composition of marine macroalgal extracts, nanoparticle formation, and the resulting biological activities. In particular, studies integrating detailed phytochemical profiling of the reducing extract with comprehensive physicochemical characterization of the synthesized nanoparticles and a direct comparison of the biological activities of both materials are still scarce. Addressing these aspects is essential for improving the understanding of the mechanisms underlying algal-mediated nanoparticle synthesis and for facilitating the rational development of reproducible and application-oriented green nanomaterials [12,13].

Although numerous studies have reported the green synthesis of silver nanoparticles using plants, microorganisms, and marine algae [2,7,14], most have primarily focused on nanoparticle synthesis or limited biological evaluation. Comprehensive studies integrating phytochemical characterization of the reducing extract, detailed physicochemical characterization of the synthesized nanoparticles, and direct comparison of the biological activities of crude algal extracts and their corresponding biosynthesized AgNPs remain scarce. Therefore, the present study aimed not only to synthesize silver nanoparticles using *C. linum* through an environmentally friendly approach but also to characterize the reducing extract by HPLC-DAD, comprehensively investigate the physicochemical properties of the synthesized AgNPs, and systematically compare their antimicrobial and antioxidant activities with those of the corresponding crude extract. This integrated strategy provides new insights into the relationship between extract composition, nanoparticle formation, and biological performance, thereby extending the current knowledge on *C. linum*-mediated green synthesis and highlighting its potential for biomedical, pharmaceutical, food safety, and environmental applications.

## 2. Material and Methods

### 2.1. Chemicals and Reagents

Silver nitrate (AgNO_3_), ethanol (95%), sodium hydroxide (NaOH), hydrochloric acid (HCl), phosphoric acid, acetonitrile (HPLC grade), methanol, DPPH, ABTS, potassium persulfate, neocuproine, copper (II) chloride, ammonium acetate, ferrozine, iron (II) chloride, sodium phosphate, ammonium molybdate, sulfuric acid, BHT, BHA, EDTA, gentamicin, fluconazole, and dimethyl sulfoxide (DMSO) were purchased from Sigma-Aldrich (St. Louis, MO, USA). The reference standards used for HPLC-DAD analysis, including catechin hydrate, caffeic acid, 4-hydroxybenzoic acid, vanillin, *p*-coumaric acid, rutin, naringin, rosmarinic acid, resveratrol, quercetin, *trans*-cinnamic acid, naringenin, chrysin, and flavone, were also obtained from Sigma-Aldrich (St. Louis, MO, USA). Mueller–Hinton agar, Mueller–Hinton broth, Sabouraud dextrose agar, and Sabouraud dextrose broth were purchased from Merck (Darmstadt, Germany). All chemicals were of analytical grade unless otherwise stated, and ultrapure/deionized water was used throughout the experiments.

### 2.2. Sample Collection

Specimens of *C. linum* were collected via scuba diving from the Yapıldakaltı shoreline located in the province of Çanakkale, Türkiye. The collected algal material was transferred to the laboratory under cold-chain conditions in sterile glass containers. In the laboratory, necrotic tissues and debris were removed by rinsing the samples with distilled water. The cleaned biomass was subsequently homogenized and stored at room temperature until further processing. A satellite view of the sampling site is presented in Figure 1. The geographic coordinates of the station are 40°14′27.03″ N and 26°32′29.74″ E.

HPLC-DAD 1260. Infinity Series, Agilent Technologies, Santa Clara, CA, USA), following a previously reported method with minor modifications [15]. Chromatographic separation was performed on an ACE Generix C18 column (250 × 4.6 mm, 5 μm particle size) maintained at 30 °C. The injection volume was 10 μL, and the flow rate was 0.8 mL min^−1^. The mobile phase consisted of acetonitrile (solvent A) and 0.1% phosphoric acid in water (solvent C). Gradient elution was carried out according to the following program: 0 min, 83% A/17% C; 7 min, 85% A/15% C; 20 min, 80% A/20% C; 24 min, 75% A/25% C; 28 min, 70% A/30% C; 30 min, 60% A/40% C; 32 min, 50% A/50% C; 36 min, 30% A/70% C; and 40 min, 83% A/17% C. Detection was performed using a diode array detector at 300 nm, with a reference wavelength of 500 nm.

Phenolic compounds were identified by comparing the retention times of sample peaks with those of authentic reference standards analyzed under identical chromatographic conditions. Quantification was performed using the external standard calibration method, and the concentrations of the identified compounds were expressed as ng/μL. Calibration curves for all reference standards exhibited excellent linearity (R^2^ > 0.99), confirming the reliability of the quantitative analysis. This chromatographic analysis was performed to characterize the phenolic constituents of the aqueous extract and to provide experimental evidence supporting the proposed biomolecule-mediated mechanism of silver nanoparticle biosynthesis by identifying the redox-active metabolites potentially involved in Ag^+^ reduction and nanoparticle stabilization. The Figure S1 was generated using ChatGPT (OpenAI) on 11 July 2026. The input prompts were designed by the authors to generate a schematic illustration of the proposed mechanism of CL-AgNP biosynthesis mediated by the aqueous extract of *Chaetomorpha linum*, illustrating the involvement of phytochemicals identified by HPLC-DAD in the reduction of Ag^+^ ions, nanoparticle nucleation and growth, and subsequent capping and stabilization of the formed AgNPs. The generated output was subsequently reviewed and formatted by the authors for appropriate presentation in the manuscript. All AI-generated content was manually reviewed and validated by the authors to ensure its scientific accuracy and consistency with the experimental data presented in the manuscript.

### 2.3. Biosynthesis of Silver Nanoparticles (CL-AgNPs)

The biosynthesis of silver nanoparticles (AgNPs) was carried out following the method described by M’sakni et al. (2023) with minor modifications [16]. Briefly, 5 g of *C. linum* biomass was mixed with hot deionized water at a ratio of 1:40 (*w*/*v*) and stirred for 2 h at 80 °C. The mixture was filtered through a 0.45 μm Millipore membrane and centrifuged at 5000 rpm for 20 min. The resulting supernatant was precipitated by adding 95% ethanol (40:60, *v*/*v*) and stored at 4 °C for 12 h. Subsequently, the solution was centrifuged again at 5000 rpm for 30 min, and the obtained *C. linum* water extract (CL-WE) fraction was resuspended in deionized water.

Prior to nanoparticle synthesis, preliminary optimization experiments were performed to determine the optimum reaction pH and temperature. The synthesis reaction was evaluated at pH 7, 9, and 11 and at 40, 50, and 60 °C, following the conditions reported by M’sakni et al. (2023) [16]. Nanoparticle formation was monitored by visual observation and UV–Vis spectroscopy. Among the tested conditions, pH 9 and 40 °C produced the most intense surface plasmon resonance (SPR) band and showed no visible aggregation during the synthesis period; therefore, these conditions were selected for subsequent experiments.

For AgNP biosynthesis, 10 mL of CL-WE was added to 90 mL of 1 mM silver nitrate (AgNO_3_) solution and incubated under continuous magnetic stirring at 40 °C for 48 h. The pH of the reaction mixture was adjusted to 9.0 by the dropwise addition of 0.1 M NaOH. To monitor nanoparticle formation, aliquots were collected at the onset of the first visible color change and after 1, 2, 4, 6, 8, 12, 24, and 48 h for UV–Vis analysis. A gradual color change from pale yellow to dark brown was observed during incubation, indicating the formation of silver nanoparticles.

Following completion of the reaction, the biosynthesized AgNPs were collected by centrifugation at 10,000 rpm for 20 min, washed three times with deionized water to remove residual silver ions and unbound biomolecules, and dried to constant weight. The dried nanoparticles were stored in sterile amber glass vials at room temperature until further physicochemical characterization and biological analyses. The recovered dried CL-AgNP product was subsequently used for all characterization and biological activity assays.

### 2.4. Characterization of AgNPs

The structural, morphological, elemental, and physicochemical characteristics of the biosynthesized AgNPs were investigated using Fourier-transform infrared spectroscopy (FTIR), X-ray diffraction (XRD), field emission scanning electron microscopy (FESEM), energy-dispersive X-ray spectroscopy (EDS), and dynamic light scattering (DLS).

### 2.5. Fourier-Transform Infrared Spectroscopy (FTIR)

FTIR spectra of the dried CL-EXT and CL-AgNP samples were recorded using a Bruker Vertex 70 Fourier-transform infrared spectrometer (Bruker Optics GmbH, Ettlingen, Germany) equipped with an attenuated total reflectance (ATR) accessory. Spectra were acquired over the range of 4000–400 cm^−1^ at a spectral resolution of 4 cm^−1^ using 32 scans per sample. FTIR analysis was performed to identify the functional groups associated with the biomolecules involved in the reduction and stabilization (capping) of the biosynthesized AgNPs [14,17].

### 2.6. X-Ray Diffraction (XRD)

The crystalline structure and phase composition of the biosynthesized CL-AgNPs were characterized by X-ray diffraction (XRD) using a PANalytical X-ray diffractometer (Malvern Panalytical, Almelo, The Netherlands) equipped with a PIXcel detector and Cu-Kα radiation (λ = 1.5406 Å). The instrument was operated at 40 kV and 30 mA. Diffraction patterns were recorded over a 2θ range of 20–80° with a step size of 0.02° and a scanning rate of 2° min^−1^. The diffraction peaks were indexed by comparison with the standard JCPDS Card No. 04-0783 to identify the crystalline phase of metallic silver. The apparent crystallite size of the biosynthesized CL-AgNPs was estimated using the Scherrer equation, $D=K\lambda /\left(\beta \cos\theta \right)$, where *D* is the apparent crystallite size, *K* is the shape factor (0.9), *λ* is the X-ray wavelength, *β* is the full width at half maximum (FWHM) of the diffraction peak expressed in radians, and *θ* is the Bragg angle [18]. As instrumental broadening could not be corrected using a standard reference material, the calculated values are reported as apparent crystallite sizes.

### 2.7. Field Emission Scanning Electron Microscopy (FESEM)

Field emission scanning electron microscopy (FESEM) was performed to examine the morphology and surface characteristics of the biosynthesized CL-AgNPs using a ZEISS GeminiSEM (Carl Zeiss AG, Oberkochen, Germany). A small amount of the dried CL-AgNP powder was mounted onto carbon adhesive tape attached to an aluminum stub and directly examined without conductive coating at an accelerating voltage of 5.0 kV and a working distance of 7 mm. Representative micrographs were acquired to evaluate the morphology, particle size, and surface characteristics of the synthesized nanoparticles.

### 2.8. Transmission Electron Microscopy (TEM)

The morphology and primary particle size of the biosynthesized CL-AgNPs were examined by transmission electron microscopy (TEM) using a JEOL JEM-1220 transmission electron microscope (JEOL Ltd., Tokyo, Japan) at the Central Research Laboratory Application and Research Center (ARUM), Eskişehir Osmangazi University, Eskişehir, Türkiye. TEM observations were performed at an accelerating voltage of 100 kV. Representative TEM micrographs were acquired to evaluate the morphology, dispersion, and primary particle dimensions of the synthesized nanoparticles. Particle size analysis was performed using ImageJ software (version 1.54r, National Institutes of Health, Bethesda, MD, USA) software by measuring eight clearly distinguishable individual nanoparticles (*n* = 8), and the results were expressed as the mean ± standard deviation (SD).

### 2.9. Energy-Dispersive X-Ray Spectroscopy (EDS)

The elemental composition and spatial distribution of the biosynthesized CL-AgNPs were determined by energy-dispersive X-ray spectroscopy (EDS) using an EDS detector coupled to the FESEM instrument. EDS spectra and elemental mapping images were acquired from representative regions of the samples to evaluate the distribution of the detected elements. The elemental composition was quantified and expressed as weight percentage (wt%).

### 2.10. Dynamic Light Scattering (DLS)

The hydrodynamic particle size distribution and polydispersity index (PDI) of the biosynthesized AgNPs were determined by dynamic light scattering (DLS) using a NanoPlus 3 Dynamic Light Scattering (DLS) analyzer (Micromeritics Instrument Corporation, Norcross, GA, USA). Measurements were performed at 25 °C using freshly prepared nanoparticle suspensions, and each sample was analyzed in triplicate. The average hydrodynamic diameter and PDI values were calculated automatically by the instrument software from the intensity-weighted particle size distributions.

### 2.11. Antimicrobial Assays

All antimicrobial assays were performed in triplicate, and inhibition zone diameters and MIC values are presented as the mean ± standard deviation (SD). Statistical comparisons between groups were performed using Student’s *t*-test, and differences were considered statistically significant at *p* < 0.05.

### 2.12. Microorganisms

Antimicrobial activity was assessed against Gram-positive bacteria (*Staphylococcus aureus* ATCC 6538 and *Bacillus subtilis* ATTC 21332), Gram-negative bacteria (*Escherichia coli* ATCC 8739, *Pseudomonas aeruginosa* ATCC 9027 and *Salmonella typhimurium* NCTC 12023), and the fungal strain *Candida albicans* ATCC 10231. Bacterial suspensions were adjusted to 0.5 McFarland standard (~10^8^ CFU/mL) prior to testing. Antimicrobial susceptibility testing was performed following the recommendations of the Clinical and Laboratory Standards Institute [19,20].

### 2.13. Disk Diffusion Assay

The antimicrobial activity of CL-AgNPs and *C. linum* extract (CL-EXT) was initially evaluated using the disk diffusion method. The assay was performed according to the Clinical and Laboratory Standards Institute (CLSI) M02 guidelines with minor modifications [20]. The bacterial and fungal suspensions were adjusted to the 0.5 McFarland standard (approximately 1.5 × 10^8^ CFU/mL for bacteria) prior to inoculation. Bacterial strains were inoculated onto Mueller–Hinton agar (MHA) plates, whereas *Candida albicans* was inoculated onto Sabouraud dextrose agar (SDA) plates using sterile cotton swabs. Sterile blank paper disks (6 mm in diameter) were impregnated with 20 μL of each test sample (6.25 mg/mL, corresponding to 125 μg/disk), allowed to dry under aseptic conditions, and then placed onto the inoculated agar surfaces. Commercial gentamicin (30 μg/disk) and fluconazole (30 μg/disk) disks were used as positive controls for the antibacterial and antifungal assays, respectively. The plates were incubated at 37 °C for 24 h, after which the diameters of the inhibition zones, including the disk diameter, were measured in millimeters (mm).

### 2.14. Broth Microdilution Assay (MIC)

Minimum inhibitory concentrations (MICs) of CL-AgNPs and *Chaetomorpha linum* extract (CL-EXT) were determined using the broth microdilution method according to the Clinical and Laboratory Standards Institute (CLSI) M07 guidelines with minor modifications. A 25 mg/mL stock solution of each sample was prepared in DMSO. Subsequently, a 6.25 mg/mL working solution was prepared from the stock solution and used as the starting concentration for two-fold serial dilutions in sterile Mueller–Hinton broth (MHB), yielding final concentrations ranging from 6.25 to 0.012 mg/mL. Bacterial suspensions were adjusted to the 0.5 McFarland standard and diluted to obtain a final inoculum of approximately 5 × 10^5^ CFU/mL in each well. Thereafter, 100 μL of each sample dilution and 100 μL of the standardized microbial suspension were dispensed into sterile 96-well U-bottom microtiter plates, resulting in a final assay volume of 200 μL per well. *Candida albicans* was evaluated under identical experimental conditions using Sabouraud dextrose broth (SDB). Gentamicin and fluconazole were used as positive controls for the antibacterial and antifungal assays, respectively, whereas DMSO served as the negative control. Microplates were incubated at 37 °C for 24 h, and the MIC was defined as the lowest concentration that completely inhibited visible microbial growth.

### 2.15. Antioxidant Assay

The antioxidant activity of CL-EXT and CL-AgNPs was evaluated using complementary in vitro assays, including DPPH radical scavenging, ABTS radical cation decolorization, Cupric Ion Reducing Antioxidant Capacity (CUPRAC), and metal chelating activity assays. Because each assay is based on a different antioxidant mechanism, their combined use provides a comprehensive assessment of antioxidant potential. Specifically, DPPH and ABTS assays evaluate free radical scavenging activity, CUPRAC measures electron-donating (reducing) capacity, and the metal chelating assay determines the ability to chelate transition metal ions involved in oxidative reactions. All assays were performed in triplicate, and the results are expressed as mean ± standard deviation (SD). IC_50_ values were calculated for the DPPH, ABTS, and metal chelating assays, whereas CUPRAC results are presented as A_0.5_ values, representing the concentration required to produce an absorbance of 0.50.

### 2.16. DPPH Radical Scavenging Assay

The DPPH radical scavenging activity was determined with slight modifications. Briefly, 40 μL of each sample solution at different concentrations was mixed with 160 μL of freshly prepared DPPH solution in a 96-well microplate and incubated in the dark at room temperature. The absorbance was measured at 517 nm using a Multiskan™ GO Microplate Spectrophotometer (Thermo Fisher Scientific, Vantaa, Finland). BHT and BHA were used as reference antioxidants. The radical scavenging activity was expressed as the percentage inhibition by comparing the absorbance of the sample with that of the control.

### 2.17. ABTS Radical Scavenging Assay

The ABTS radical cation scavenging assay was performed with slight modifications. The ABTS•^+^ radical cation was generated by reacting ABTS with potassium persulfate and incubating the mixture in the dark. Subsequently, 40 μL of the sample solution was mixed with 160 μL of freshly prepared ABTS•^+^ working solution in a 96-well microplate. After incubation at room temperature, the absorbance was measured at 734 nm using a microplate reader. BHT and BHA were used as reference antioxidants, and the antioxidant activity was expressed as the percentage inhibition relative to the control.

### 2.18. Cupric Ion Reducing Antioxidant Capacity (CUPRAC) Assay

The cupric ion reducing antioxidant capacity (CUPRAC) assay was performed according to the method described by Apak et al. (2004) with slight modifications [21]. Briefly, 40 μL of the sample solution was mixed with 50 μL of copper (II) chloride solution, 50 μL of neocuproine solution, and 60 μL of ammonium acetate buffer solution in a 96-well microplate. Following incubation at room temperature, the absorbance was measured at 450 nm using a microplate reader. BHT and BHA were used as reference antioxidants. The antioxidant capacity of the samples was expressed as Trolox equivalent antioxidant capacity (TEAC, mmol Trolox g^−1^) using a Trolox calibration curve.

### 2.19. Metal Chelating Activity

The metal chelating activity was determined according to the method with slight modifications. Briefly, 40 μL of the sample solution was mixed with 40 μL of ethanol, 40 μL of 0.2 mM FeCl_2_ solution, and 80 μL of 0.5 mM ferrozine solution in a 96-well microplate. Following incubation at room temperature, the absorbance was measured at 593 nm using a microplate reader. EDTA was used as the positive control. The metal chelating activity was expressed as the percentage inhibition relative to the control.

### 2.20. Statistical Analysis

All experiments were performed in triplicate, and the results are expressed as the mean ± standard deviation (SD). Statistical analyses were performed using Student’s *t*-test to compare differences between experimental groups. Differences were considered statistically significant at *p* < 0.05. All statistical analyses were carried out using GraphPad Prism version 8.0.2 (GraphPad Software, San Diego, CA, USA).

## 3. Results

### 3.1. UV–Visible Spectroscopic Analysis of Biosynthesized CL-AgNPs

The optimized biosynthesis procedure yielded 18.9 mg of dried CL-AgNPs. The formation of silver nanoparticles was initially indicated by a gradual color change of the reaction mixture from pale yellow to dark brown during the 48 h incubation period, reflecting the reduction of Ag^+^ ions under alkaline conditions (pH 9).

The progress of CL-AgNP biosynthesis was visually monitored through the gradual color change of the reaction mixture over time (Figure 2). Initially, the mixture exhibited a pale yellow color (Figure 2a), which progressively changed to light brown during the early stages of the reaction (Figure 2b–d) and eventually developed into a dark brown suspension after 48 h (Figure 2j). This characteristic color transition is attributed to the reduction of Ag^+^ ions to metallic silver (Ag^0^) by bioactive compounds present in the CL-EXT, leading to the formation of silver nanoparticles. The gradual increase in color intensity reflects the continuous nucleation and growth of AgNPs throughout the reaction period. Similar time-dependent color changes have been widely reported during the green synthesis of silver nanoparticles using marine macroalgal extracts and are considered a preliminary visual indicator of successful nanoparticle formation prior to spectroscopic characterization.

This visual observation was further confirmed by UV–Vis spectroscopy, which exhibited a distinct surface plasmon resonance (SPR) absorption band centered at approximately 438 nm (Figure 3). The observed SPR peak is characteristic of silver nanoparticles and is consistent with previously reported values for marine algae-mediated AgNPs, thereby confirming the successful biosynthesis of CL-AgNPs.

### 3.2. HPLC-DAD Analysis of the Phenolic Composition of CL-EXT

To further characterize the chemical composition of the aqueous extract used for AgNP biosynthesis, the phenolic profile of C. linum was analyzed by HPLC-DAD. The representative HPLC-DAD chromatogram of the aqueous extract is shown in Figure 4, while the identified phenolic compounds together with their retention times and concentrations are summarized in Table 2.

The chromatographic analysis revealed the presence of several phenolic acids and flavonoids, including catechin hydrate, caffeic acid, 4-hydroxybenzoic acid, vanillin, *p*-coumaric acid, rutin, naringin, rosmarinic acid, resveratrol, quercetin, *trans*-cinnamic acid, naringenin, chrysin, and flavones. Among the identified compounds, chrysin was the predominant constituent (821.65 ng/µL), followed by rutin (10.06 ng/µL), naringin (6.17 ng/µL), flavones (5.06 ng/µL), vanillin (4.66 ng/µL), naringenin (4.44 ng/µL), and 4-hydroxybenzoic acid (3.58 ng/µL). Lower concentrations of catechin hydrate, caffeic acid, *p*-coumaric acid, rosmarinic acid, resveratrol, quercetin, and *trans*-cinnamic acid were also detected.

The detected compounds belong predominantly to phenolic acids and flavonoids, which represent major classes of bioactive metabolites in marine macroalgae. The presence of these structurally diverse metabolites provides a comprehensive chemical characterization of the aqueous extract and establishes an experimental basis for interpreting the biomolecule-mediated biosynthesis of silver nanoparticles, which is discussed in the following section.

### 3.3. Fourier-Transform Infrared (FTIR) Analysis of CL-AgNPs

The FTIR spectra of CL-EXT and biosynthesized CL-AgNPs revealed several characteristic absorption bands and slight shifts following nanoparticle formation, indicating the involvement of algal biomolecules in the synthesis and stabilization processes (Figure 5). The broad absorption band observed at 3357 cm^−1^ in CL-EXT shifted to 3292 cm^−1^ in CL-AgNPs, corresponding to O–H stretching vibrations of phenolic compounds and alcohols, suggesting the participation of hydroxyl groups in the reduction and capping of AgNPs. Similarly, the band at 1636 cm^−1^ in CL-EXT shifted to 1639 cm^−1^ in CL-AgNPs, which can be attributed to C=O stretching (amide I) and aromatic C=C vibrations, indicating the involvement of proteins and phenolic constituents during nanoparticle synthesis. The bands observed at 1404 cm^−1^ were associated with O–H bending and C–N stretching vibrations, while those detected at 1225, 1140, and 1025 cm^−1^ were assigned to C–O and C–O–C stretching vibrations of alcohols, ethers, and polysaccharides [22,23].

In addition, the band observed around 993 cm^−1^ was attributed to polysaccharide-related vibrations, whereas the band at 823 cm^−1^ was associated with aromatic C–H out-of-plane bending and sulfated polysaccharides. The observed shifts and persistence of these functional groups suggest that the phytochemicals present in *C. linum* extract act as both reducing and stabilizing agents during the green synthesis of AgNPs.

### 3.4. X-Ray Diffraction (XRD) Analysis of CL-AgNPs

The crystalline structure of the biosynthesized CL-AgNPs was confirmed by X-ray diffraction (XRD) analysis (Figure 6). The diffraction pattern exhibited four characteristic reflections at 2θ values of approximately 38.1°, 44.3°, 64.5°, and 77.4°, corresponding to the (111), (200), (220), and (311) crystallographic planes of metallic silver, respectively. These reflections are in excellent agreement with the standard JCPDS Card No. 04-0783, confirming the formation of a face-centered cubic (fcc) crystal structure of metallic silver. In addition to the principal reflections, several weak diffraction features with low intensities were observed at higher diffraction angles. Owing to their low intensities, these reflections could not be unequivocally assigned to crystalline silver and are likely associated with residual biomolecular capping constituents and/or trace crystalline impurities originating from the green synthesis process [13,24].

The crystallite size, estimated using the Scherrer equation, was approximately 14.8 nm for the most intense (111) reflection [18]. When the four principal reflections ((111), (200), (220), and (311)) were considered, the average apparent crystallite size was 13.3 ± 3.3 nm. Since instrumental broadening could not be corrected using a standard reference material, these values are reported as apparent crystallite sizes. Collectively, these findings confirm the successful biosynthesis of highly crystalline CL-AgNPs with a well-defined face-centered cubic (fcc) silver structure [25].

### 3.5. Field Emission Scanning Electron Microscopy (FESEM) Analysis of CL-AgNPs

FESEM analysis revealed that the biosynthesized AgNPs were predominantly spherical and relatively well dispersed, although limited aggregation was observed in some regions (Figure 7). Particle size analysis was performed using ImageJ software by measuring 50 well-isolated primary nanoparticles, while aggregated regions were excluded from the analysis.

The synthesized AgNPs exhibited an average particle diameter of 31.09 ± 2.52 nm (*n* = 50), with particle sizes ranging from 21.26 to 42.90 nm. These results confirm the successful formation of nanoscale silver nanoparticles with a relatively narrow particle size distribution. A limited degree of particle aggregation was also observed in the FESEM micrographs. The nanoscale dimensions and predominantly spherical morphology further confirm the successful biosynthesis of AgNPs using *C. linum* extract [26].

### 3.6. Transmission Electron Microscopy (TEM) Analysis of CL-AgNPs

Transmission electron microscopy (TEM) analysis revealed that the biosynthesized CL-AgNPs were predominantly spherical to quasi-spherical in morphology, with mostly discrete primary nanoparticles and limited particle association (Figure 8). Particle size analysis was performed using ImageJ software on eight clearly distinguishable individual nanoparticles (*n* = 8), yielding an average particle diameter of 30.87 ± 2.94 nm. This value was in close agreement with the average particle diameter obtained by FESEM (31.09 ± 2.52 nm, *n* = 50), confirming the consistency of the primary particle size measurements obtained by the two complementary electron microscopy techniques.

### 3.7. Energy-Dispersive X-Ray Spectroscopy (EDS) Analysis of CL-AgNPs

Energy-dispersive X-ray spectroscopy (EDS) was performed to determine the elemental composition of the biosynthesized AgNPs (Figure 9). The EDS spectrum confirmed the presence of silver as the predominant element, accounting for 91.4 wt%, thereby verifying the successful formation of silver nanoparticles. In addition to Ag, carbon (6.5 wt%), oxygen (1.9 wt%), and a trace amount of chlorine (0.3 wt%) were also detected. The corresponding elemental maps revealed the qualitative spatial distribution of Ag, C, and O within the analyzed region. These results confirm the elemental composition of the biosynthesized AgNPs, while the detected carbon- and oxygen-containing elements are consistent with the presence of algal-derived biomaterial associated with the nanoparticles [27].

### 3.8. Dynamic Light Scattering (DLS) Analysis of CL-AgNPs

The hydrodynamic size distribution of the biosynthesized CL-AgNPs was determined by dynamic light scattering (DLS), and the cumulative intensity distribution curves are presented in Figure 10. The three independent measurements exhibited highly similar distribution profiles, indicating good reproducibility of the measurements. The mean hydrodynamic diameter was 646.5 ± 18.9 nm (mean ± SD, *n* = 3) (Figure 10). The corresponding particle size distribution percentiles were D10 = 295.0 ± 6.1 nm, D50 = 557.9 ± 12.8 nm, and D90 = 1062.6 ± 47.9 nm (mean ± SD, *n* = 3), indicating that 50% of the particle population exhibited hydrodynamic diameters below approximately 558 nm. The relatively broad distribution range suggests the presence of particle aggregates and biomolecule-coated nanoparticle clusters in the colloidal suspension. The polydispersity index (PDI) values ranged between 0.219 and 0.245, with an average value of 0.233, indicating moderate polydispersity of the nanoparticle suspension. The low variation among replicate measurements further confirms the reproducibility of the DLS measurements.

The DLS results indicate that the biosynthesized CL-AgNPs formed a colloidally stable nanoparticle suspension with moderate polydispersity and hydrodynamic diameters in the submicron range. The high similarity among replicate measurements confirms the reproducibility of the synthesis process and the uniformity of the resulting nanoparticle dispersion.

In addition, the original instrumental DLS output showing the particle size distribution by normalized intensity for the three independent measurements is presented in Figure 11. The highly overlapping intensity distribution profiles provide direct visualization of the reproducibility of the DLS measurements and the hydrodynamic size distribution of the CL-AgNP suspension.

### 3.9. Antimicrobial Activity

Antimicrobial susceptibility was evaluated using disk diffusion and broth microdilution assays.

### 3.10. Disk Diffusion Assay of CL-AgNPs

Disk diffusion analysis demonstrated distinct differences in antimicrobial activity between CL-EXT, CL-AgNP and gentamicin (Table 3; Figure 12). CL-AgNP generated measurable inhibition zones against *S. aureus* (13 mm), *B. subtilis* (10 mm) and *S. typhimurium* (0.7 mm), whereas no inhibition was observed against *E. coli*, *P. aeruginosa* or *C. albicans* under the tested conditions. CL-EXT displayed limited antibacterial activity, forming a 0.8 mm inhibition zone only against *B. subtilis*. In contrast, gentamicin exhibited strong activity against all tested bacterial strains, with inhibition zones ranging from 21 to 42 mm. None of the tested agents produced inhibition zones against *C. albicans*.

### 3.11. Broth Microdilution Assay (MIC) of CL-AgNPs

Broth microdilution assays further showed that CL-AgNP exhibited markedly higher antimicrobial potency than CL-EXT (Table 4). CL-AgNP produced a MIC value of 1.56 μg/mL against *S. aureus* ATCC 6538, *E. coli* ATCC 8739 and *C. albicans* ATCC 10231. CL-EXT displayed antimicrobial activity only against *E. coli*, with a MIC value of 12.5 μg/mL. No detectable MIC was observed for CL-EXT against the remaining test strains.

The antimicrobial profiles obtained via the disk diffusion and broth microdilution assays revealed distinct differences between the extract and nanoparticle formulations. CL-AgNPs exhibited antimicrobial activity against a broader range of test microorganisms than CL-EXT. While inhibition zones were primarily observed against Gram-positive bacteria in the disk diffusion assay, the broth microdilution assay demonstrated inhibitory activity of CL-AgNPs against Gram-positive bacteria, Gram-negative bacteria, and *C. albicans*. This difference can be attributed to the distinct principles of the two methods, as the disk diffusion assay depends on the diffusion of the antimicrobial agent through the agar matrix, whereas the broth microdilution assay evaluates microbial growth inhibition under direct contact in a liquid medium. Because silver nanoparticles may exhibit limited diffusion in agar due to their particle size and aggregation behavior, the absence of inhibition zones does not necessarily indicate the absence of antimicrobial activity. In contrast, CL-EXT displayed limited antimicrobial activity, with measurable effects observed only against *B. subtilis* in the disk diffusion assay and *E. coli* in the broth microdilution assay. The MIC values of the positive controls were within the expected ranges for the tested reference microorganisms, with gentamicin exhibiting the highest antibacterial activity against the bacterial strains and fluconazole serving as the positive antifungal control against *C. albicans*. These findings support the reliability of the antimicrobial susceptibility assays and confirm the enhanced antimicrobial efficacy of CL-AgNPs compared with the crude extract [28].

### 3.12. Antioxidant Activity

The antioxidant activity profiles of *Chaetomorpha linum* extract (CL-EXT) and silver nanoparticles (CL-AgNPs) were evaluated using DPPH, ABTS, CUPRAC, and metal chelating assays (Table 5). In radical scavenging assays, lower IC_50_ values indicate stronger antioxidant capacity, whereas higher absorbance values in the CUPRAC assay reflect greater reducing power.

CL-AgNPs exhibited superior antioxidant activity compared with the crude extract across all tested assays. In the DPPH assay, CL-AgNPs achieved a radical scavenging activity of 78.65 ± 0.98% with an IC_50_ value of 24.67 ± 0.43 µg/mL, whereas CL-EXT showed 56.34 ± 0.21% inhibition and an IC_50_ value of 32.40 ± 0.18 µg/mL. A similar trend was observed in the ABTS assay, where CL-AgNPs demonstrated 80.12 ± 0.13% inhibition and a lower IC_50_ value (17.85 ± 0.62 µg/mL) than CL-EXT (65.77 ± 0.26%, IC_50_ = 29.80 ± 0.24 µg/mL). These findings indicate that nanoparticle formation enhanced the radical scavenging efficiency of *C. linum*-derived bioactive compounds. The reducing power determined by the CUPRAC assay further supported this enhancement. CL-AgNPs exhibited a higher absorbance value (4.74 ± 0.02) and a lower A_0.50_ value (28.87 ± 0.91 µg/mL) than CL-EXT (3.18 ± 0.04 and 35.60 ± 0.22 µg/mL, respectively), indicating a greater electron-donating capacity. Likewise, metal chelating activity was enhanced following nanoparticle synthesis. CL-AgNPs displayed 362.35 ± 0.24% inhibition with an IC_50_ value of 31.93 ± 0.47 µg/mL, whereas CL-EXT showed 286.88 ± 0.48% inhibition and an IC_50_ value of 45.30 ± 0.35 µg/mL.

When compared with the synthetic antioxidant standards (BHT, BHA, and EDTA), both CL-EXT and CL-AgNPs exhibited considerable antioxidant activity, although the standards generally showed stronger radical scavenging and reducing capacities. Nevertheless, the relatively low IC_50_ and A_0.50_ values obtained for CL-AgNPs indicate a substantial antioxidant potential. The enhanced antioxidant performance of the nanoparticles may be attributed to the synergistic interaction between silver nanostructures and surface-bound algal metabolites, including phenolic compounds, polysaccharides, and other redox-active constituents that remained associated with the nanoparticle surface during biosynthesis.

## 4. Discussion

The growing limitations of conventional nanoparticle synthesis, particularly the extensive use of hazardous chemicals and energy-intensive processes, have increased interest in green nanotechnology as a sustainable alternative. Marine macroalgae have emerged as promising biological platforms for nanoparticle biosynthesis owing to their intrinsic reducing and capping metabolites. In this context, the present findings further demonstrate that *Chaetomorpha linum* is a valuable marine bioresource for the environmentally friendly production of biofunctional AgNPs [7,29].

Morphological and spectroscopic analyses confirmed the successful biosynthesis of CL-AgNPs. The characteristic surface plasmon resonance band at 438 nm provided initial evidence of nanoparticle formation and was consistent with previous reports on algae-mediated AgNP synthesis [14]. To further elucidate the biomolecule-mediated mechanism of AgNP biosynthesis, the aqueous *C. linum* extract was characterized by HPLC-DAD analysis. The chromatographic analysis identified several phenolic acids and flavonoids, including catechin hydrate, caffeic acid, 4-hydroxybenzoic acid, vanillin, *p*-coumaric acid, rutin, naringin, rosmarinic acid, resveratrol, quercetin, trans-cinnamic acid, naringenin, chrysin, and flavones. These redox-active metabolites are well known to promote Ag^+^ reduction and nanoparticle stabilization during green synthesis [12,14,17,30].

FTIR analysis further confirmed the presence of functional groups associated with biomolecules involved in nanoparticle reduction and capping, whereas XRD analysis verified the crystalline face-centered cubic structure of the biosynthesized AgNPs through the characteristic (111), (200), (220), and (311) reflections.

The HPLC-DAD findings were further supported by FTIR analysis, which revealed characteristic absorption bands corresponding to hydroxyl (3292 cm^−1^), carbonyl/amide (1639 cm^−1^), amine (1404 cm^−1^), C–O (1225 and 1025 cm^−1^), and polysaccharide-associated vibrations (993 cm^−1^). Together, these results indicate that phenolic compounds, polysaccharides, proteins, and other oxygen-containing biomolecules are the principal reducing and stabilizing constituents of the aqueous extract, consistent with previous reports on algae-mediated AgNP biosynthesis [13,14].

Based on the combined HPLC-DAD and FTIR results [12,14,31], a plausible mechanism for AgNP biosynthesis can be proposed. The proposed biomolecule-mediated mechanism of CL-AgNP biosynthesis based on the HPLC-DAD and FTIR findings is schematically illustrated in Supplementary Figure S1. Phenolic acids and flavonoids identified in the aqueous extract donate electrons to Ag^+^ ions, promoting their reduction to metallic Ag^0^, followed by nucleation and crystal growth. Subsequently, hydroxyl-, carbonyl-, ether-, amino-, and sulfate-containing biomolecules adsorb onto the nanoparticle surface, forming a bioorganic capping layer that minimizes aggregation and enhances colloidal stability [14,17,31].

The successful biosynthesis of CL-AgNPs under mildly alkaline conditions (pH 9) is consistent with previous reports demonstrating that polysaccharides, phenolics, proteins, and reducing sugars present in algal extracts simultaneously function as reducing and capping agents. Alkaline conditions promote the deprotonation of hydroxyl, carboxyl, and amino groups, facilitating electron transfer to Ag^+^ ions while improving nanoparticle stabilization and size uniformity. This mechanism is characteristic of algae-mediated green synthesis and has been widely reported for biologically synthesized AgNPs [2,27].

FESEM analysis demonstrated predominantly spherical nanoparticles with an average particle diameter of 31.09 ± 2.52 nm (*n* = 50), ranging from 21.26 to 42.90 nm. TEM observations further confirmed discrete primary nanoparticles with an average diameter of 30.87 ± 2.94 nm (*n* = 8), in excellent agreement with the FESEM measurements. In contrast, DLS analysis yielded a substantially larger mean hydrodynamic diameter of 646.5 ± 18.9 nm (*n* = 3); a phenomenon commonly reported for biologically synthesized nanoparticles because DLS measures particles in suspension together with their biomolecular capping layer, hydration shell, adsorbed solvent molecules, and soft aggregates, whereas FESEM and TEM determine the physical diameter of the dried metallic core [32].

Therefore, the considerably larger DLS values are best explained by the combined influence of the biomolecular capping layer, hydration-associated solvent molecules, and partial nanoparticle aggregation rather than by the actual size of the metallic nanoparticle core. Moreover, the relatively low PDI value (0.233 ± 0.013) and the excellent agreement among three independent measurements indicate that the nanoparticle suspension remained moderately homogeneous despite the presence of these larger hydrodynamic assemblies [32].

The larger structures observed in some FESEM micrographs correspond to aggregates formed during sample drying rather than individual nanoparticles. Accordingly, particle size analysis was performed exclusively on well-dispersed primary nanoparticles, providing a representative average particle diameter of 31.09 ± 2.52 nm (21.26–42.90 nm).

The normalized DLS intensity distribution further confirmed the reproducibility of the measurements and supported a relatively narrow hydrodynamic size distribution. Although TEM demonstrated predominantly well-dispersed primary nanoparticles, zeta potential analysis would provide additional information on colloidal stability and help further distinguish the relative contributions of biomolecular capping and particle aggregation.

The limited aggregation observed in the FESEM micrographs is likely attributable to the high surface energy of nanoparticles together with the presence of biomolecules acting as capping agents during biosynthesis. Such aggregation is commonly observed in biologically synthesized AgNPs and may contribute to colloidal stability by promoting the formation of a biomolecule-rich surface corona [24]. Moreover, the nanoscale dimensions of the synthesized AgNPs provide a high surface-area-to-volume ratio, which may facilitate interactions with microbial cell membranes and thereby contribute to the enhanced antimicrobial activity observed in this study [33,34].

The smaller crystallite size compared with the particle diameters determined by FESEM (31.09 ± 2.52 nm) and TEM (30.87 ± 2.94 nm) indicates that individual nanoparticles may consist of one or more coherent crystalline domains, whereas the larger particle dimensions obtained by electron microscopy represent the overall nanoparticle size rather than the size of a single crystallite. Such differences are commonly observed because XRD determines the size of coherent crystalline domains, whereas FESEM and TEM measure the overall dimensions of individual nanoparticles; therefore, these techniques provide complementary structural information [34,35].

The polysaccharides, sulfate-containing compounds, proteins, and other metabolites identified by FTIR and EDS analyses likely contributed to the formation of a stabilizing bioorganic corona surrounding the nanoparticles, thereby increasing their hydrodynamic size. The proposed bioorganic corona is further supported by the HPLC-DAD results, which revealed multiple phenolic compounds capable of interacting with the nanoparticle surface through hydroxyl and carbonyl functionalities. Together, these findings indicate that phenolic compounds and other biomolecules act synergistically in Ag^+^ reduction and nanoparticle stabilization, consistent with previous reports on algae-mediated AgNP biosynthesis [13,14].

It should be noted that the phytochemical composition of marine macroalgae is influenced by environmental conditions, including geographical location, season, temperature, salinity, light intensity, and nutrient availability, which may consequently affect nanoparticle biosynthesis. Because all experiments were performed using a single batch of *C. linum* collected from one location during a single sampling period, seasonal and geographical variations were beyond the scope of the present study. Nevertheless, the HPLC-DAD characterization of the extract used for nanoparticle synthesis provides direct experimental support for the proposed biomolecule-mediated mechanism in the investigated sample. Future studies evaluating different collection sites and seasons would further clarify the influence of environmental variability on green AgNP biosynthesis [30,36].

In addition to their antimicrobial properties, the antioxidant activities of CL-EXT and CL-AgNPs were evaluated using DPPH, ABTS, CUPRAC, and metal chelating assays. Because oxidative stress plays a central role in numerous pathological conditions, multifunctional nanomaterials possessing both antimicrobial and antioxidant properties have attracted considerable interest [37,38]. The present results demonstrated that both CL-EXT and CL-AgNPs exhibited antioxidant activity, whereas nanoparticle formation markedly enhanced the antioxidant potential of *C. linum*.

The DPPH and ABTS assays revealed a pronounced improvement in radical scavenging activity following nanoparticle synthesis. CL-AgNPs exhibited DPPH and ABTS inhibition values of 78.65% and 80.12%, respectively, compared with 56.34% and 65.77% for CL-EXT. Likewise, the lower IC_50_ values of CL-AgNPs for DPPH (24.67 µg/mL) and ABTS (17.85 µg/mL), relative to CL-EXT (32.40 and 29.80 µg/mL, respectively), indicate greater free-radical scavenging efficiency. These findings suggest that nanoparticle formation enhances electron transfer while preserving redox-active biomolecules derived from the algal extract [30,36]. Moreover, this observation supports a clear structure–activity relationship in which the phenolic-rich biomolecular corona identified by HPLC-DAD and FTIR not only stabilizes the nanoparticle surface but also preserves the accessibility of redox-active functional groups, thereby enhancing electron transfer efficiency and antioxidant performance [39,40].

The CUPRAC assay further demonstrated the enhanced reducing capacity of CL-AgNPs. The nanoparticles exhibited a higher absorbance (4.74) and lower A_0.50_ value (28.87 µg/mL) than CL-EXT (3.18 and 35.60 µg/mL, respectively), indicating stronger electron-donating ability. These findings suggest that nanoparticle synthesis improved the accessibility and effectiveness of antioxidant constituents associated with the algal matrix [21].

Similarly, CL-AgNPs exhibited higher metal chelating activity (362.35%) and a lower IC_50_ value (31.93 µg/mL) than CL-EXT (286.88%; IC_50_ = 45.30 µg/mL). This enhanced activity may result from the synergistic interaction between the silver nanostructure and surface-bound hydroxyl, carbonyl, sulfate, and amine groups identified by FTIR analysis, which facilitate metal ion complexation [13,14].

Comparable antioxidant activities have been reported for *Chaetomorpha* species and algae-mediated AgNPs. Previous studies attributed these effects to the stabilization and concentration of phenolic and polysaccharide-rich biomolecules on the nanoparticle surface [41,42]. Consistent with these reports, the present findings demonstrate that *C. linum*-mediated AgNPs possess greater antioxidant activity than the corresponding crude extract.

The coexistence of enhanced antioxidant and antimicrobial activities highlights the potential of CL-AgNPs as multifunctional nanomaterials for biomedical, pharmaceutical, nutraceutical, food preservation, and antimicrobial coating applications [13,38].

Consistent with the disk diffusion and MIC results, CL-AgNPs exhibited markedly greater antimicrobial activity than the crude extract, indicating that nanoparticle formation enhanced the bioactivity of *C. linum*-derived metabolites. The broader antimicrobial activity observed by the broth microdilution assay compared with the disk diffusion method reflects the different principles of these techniques. Unlike agar diffusion assays, broth microdilution evaluates antimicrobial activity under direct contact in a liquid environment. Because AgNPs may diffuse poorly through agar owing to their particle size, aggregation behavior, and interactions with the agar matrix, the absence of inhibition zones does not necessarily indicate a lack of antimicrobial activity [28].

Comparable antimicrobial activities have been reported for other Chaetomorpha-mediated AgNPs. Kannan et al. (2013), Roy and Anantharaman (2017), and Al-Zahrani et al. (2021) likewise demonstrated broad-spectrum antimicrobial activity, attributing their findings to the nanoscale dimensions, spherical morphology, crystalline structure, and surface functionalization of biosynthesized AgNPs [8,17,43]. Similarly, extract-based studies have shown that *C. linum* possesses valuable antibacterial metabolites [44], while more recent investigations further highlighted its antioxidant, antimicrobial, anticancer, and multifunctional nanobiotechnological potential [41,42,45]. Collectively, these findings identify *C. linum* as a promising marine macroalga for green nanomaterial production.

The present findings further extend previous reports by demonstrating that CL-AgNPs exhibited a broader antimicrobial spectrum than CL-EXT, including an eight-fold reduction in the MIC against *Escherichia coli*. Such enhancement is unlikely to arise from a single mechanism but rather from the synergistic interplay between nanoparticle size, spherical morphology, crystalline structure, the phenolic-rich biomolecular corona, increased surface reactivity, sustained Ag^+^ release, and ROS-mediated cellular responses [35,39,46,47,48]. Together, these physicochemical characteristics promote efficient interactions with microbial cell membranes, facilitate sustained Ag^+^ release, and enhance oxidative stress induction, thereby improving the antimicrobial efficacy of CL-AgNPs compared with the crude extract [7,13,47].

From an application perspective, biosynthesized AgNPs offer several advantages over conventional antimicrobial agents because their multimodal mechanisms of action—including membrane disruption, ROS generation, protein denaturation, and interference with DNA replication—may reduce the likelihood of resistance development [46,47]. Combined with the sustainable utilization of marine biomass, these characteristics support the potential application of CL-AgNPs in wound dressings, medical coatings, food-packaging materials, disinfectants, and environmental sanitation technologies [2,5,13].

Importantly, the potential cytotoxicity of CL-AgNPs toward normal cells was evaluated using L929 fibroblasts. CL-AgNPs exhibited relatively low cytotoxicity under the investigated in vitro conditions, with cell viability remaining above 80% across the tested concentration range (0–1000 µg/mL) and exposure periods (1–24 h). The cytotoxicity of AgNPs is known to depend strongly on physicochemical characteristics such as particle size, surface chemistry, coating, aggregation state, cellular uptake, and Ag^+^ release. The relatively low cytotoxicity observed for CL-AgNPs may therefore be partly associated with their biogenic surface chemistry. HPLC-DAD and FTIR analyses indicated the involvement of algal-derived phenolic compounds, polysaccharides, proteins, and other functional biomolecules in nanoparticle capping and stabilization. Such a biomolecular coating may modulate nanoparticle–cell interactions and potentially influence Ag^+^ release and cellular internalization, thereby limiting cellular exposure to toxic silver species. Moreover, the substantially larger hydrodynamic size observed by DLS compared with the primary particle sizes determined by FESEM and TEM suggests the presence of hydrated biomolecular coatings and partial nanoparticle association, which may further influence cellular uptake. Similar studies have demonstrated that surface coating, aggregation state, cellular uptake, and silver ion release are important determinants of AgNP cytotoxicity. Nevertheless, because Ag^+^ release, cellular uptake, and intracellular ROS generation were not directly measured in the present study, these mechanisms should be regarded as plausible explanations rather than definitive conclusions. Furthermore, the present findings are limited to an in vitro L929 fibroblast model; therefore, additional studies using other normal cell types and in vivo models are required before the safety of CL-AgNPs at the tissue or organism level can be established.

The present findings further highlight *C. linum* as a promising marine resource for sustainable nanosilver production with multifunctional antioxidant and antimicrobial properties. Nevertheless, additional studies are required to evaluate activity against multidrug-resistant (MDR) pathogens, elucidate antimicrobial mechanisms through ROS generation and membrane permeability assays, further assess biosafety using additional normal cell types and in vivo models and environmental fate [13,46,47]. Further optimization of nanoparticle size, morphology, and capping chemistry may also improve biological performance and colloidal stability [14,32].

The reported yield represents the total dried CL-AgNP product recovered after biosynthesis and should not be interpreted as the Ag^+^ conversion efficiency, since the recovered material contains both silver nanoparticles and surface-associated biomolecules. Determination of the actual silver ion conversion efficiency would require quantitative analysis of residual Ag^+^ (e.g., ICP-OES or AAS), which was beyond the scope of this study.

It should be acknowledged that the biomolecular corona may influence colorimetric antioxidant assays to some extent. However, because CL-EXT and CL-AgNPs were evaluated under identical experimental conditions, the enhanced antioxidant activity is considered to primarily reflect nanoparticle formation rather than analytical interference, although potential contributions of the biomolecular corona should be considered when interpreting these assays [39,40].

Although marine macroalgae provide a scalable biomass source for green nanotechnology, seasonal, environmental, and geographical variations may influence extract composition and, consequently, nanoparticle yield and biological performance. Therefore, process standardization, quality-control protocols, batch-to-batch validation, and techno-economic evaluation will be essential for future industrial implementation [9,49,50].

Overall, the present study demonstrates that the enhanced biological performance of CL-AgNPs is governed not only by the silver core but also by the synergistic effects of particle size, morphology, surface chemistry, and the phytochemical composition of the biomolecular corona. By integrating HPLC-DAD, FTIR, XRD, FESEM, TEM, DLS, antioxidant, and antimicrobial analyses, this work establishes a comprehensive structure–activity framework that supports the development of sustainable, multifunctional AgNPs for future biomedical and environmental applications [7,42]. There are also several important works conducted in the same fields and reported in the literature [51,52,53,54,55].

## 5. Conclusions

In conclusion, this study demonstrates that *C. linum* represents a promising marine bioresource for the green synthesis of biofunctional AgNPs with broad-spectrum antimicrobial activity. The biosynthesized nanosilver exhibited inhibitory effects against both bacterial and fungal pathogens, supporting its relevance in biomedical and environmental disinfection applications. The use of macroalgal biomass provides an eco-friendly and economically feasible alternative to conventional nanoparticle synthesis, contributing to sustainable nanobiotechnology and the emerging circular bioeconomy. Given the growing need for new antimicrobial strategies in the context of AMR, marine-derived nanomaterials such as CL-AgNPs hold translational potential across pharmaceutical, clinical, food safety and bioprotective sectors. Further studies investigating biosafety, cytocompatibility and performance against MDR strains will be essential to advance *C. linum*-based nanosilver toward real-world implementation.

This study contributes to the current knowledge on *Chaetomorpha linum*-mediated silver nanoparticles by providing a comprehensive physicochemical and biological evaluation of the synthesized nanoparticles. Rather than focusing solely on the green synthesis of silver nanoparticles, this work integrates detailed structural characterization (UV–Vis, FTIR, XRD, FESEM, EDS, and DLS) with extensive antimicrobial and antioxidant evaluations. Moreover, the biological activities of the biosynthesized AgNPs were directly compared with those of the corresponding crude algal extract, demonstrating the enhancement in biological performance resulting from nanoparticle formation. Collectively, these findings provide a more comprehensive understanding of the relationship between nanoparticle characteristics and biological activity, while highlighting the potential of *C. linum*-derived AgNPs for biomedical, pharmaceutical, food safety, and environmental applications.

## Figures and Tables

**Figure 1:**
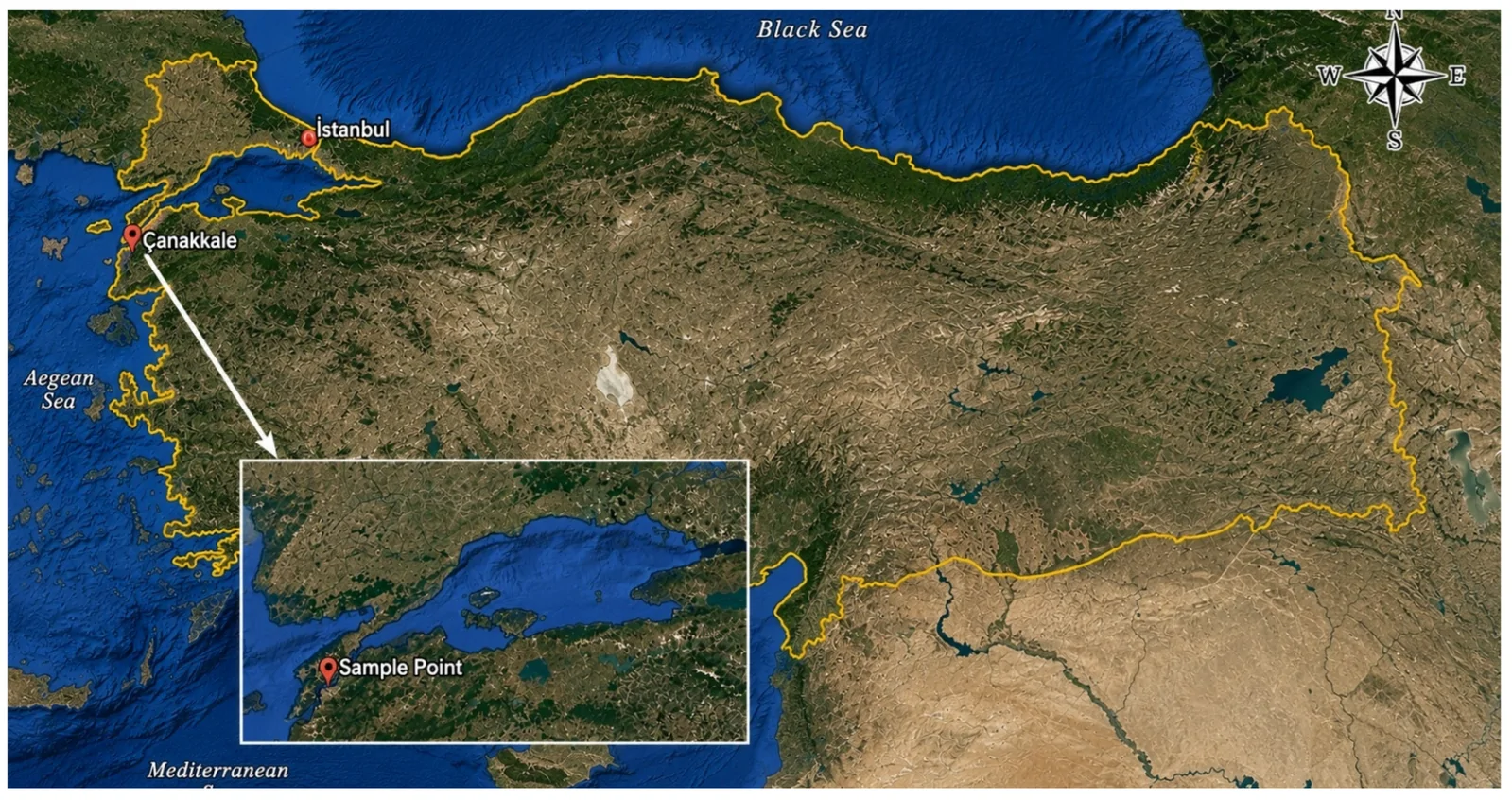
The geographic location of the sampling site (Yapıldakaltı shoreline, Çanakkale, Türkiye). The red marker indicates the collection station; Photo was created using Google Earth Pro (Version 7.3.7.1155, 64-bit; Google LLC, 2026).

**Figure 2:**
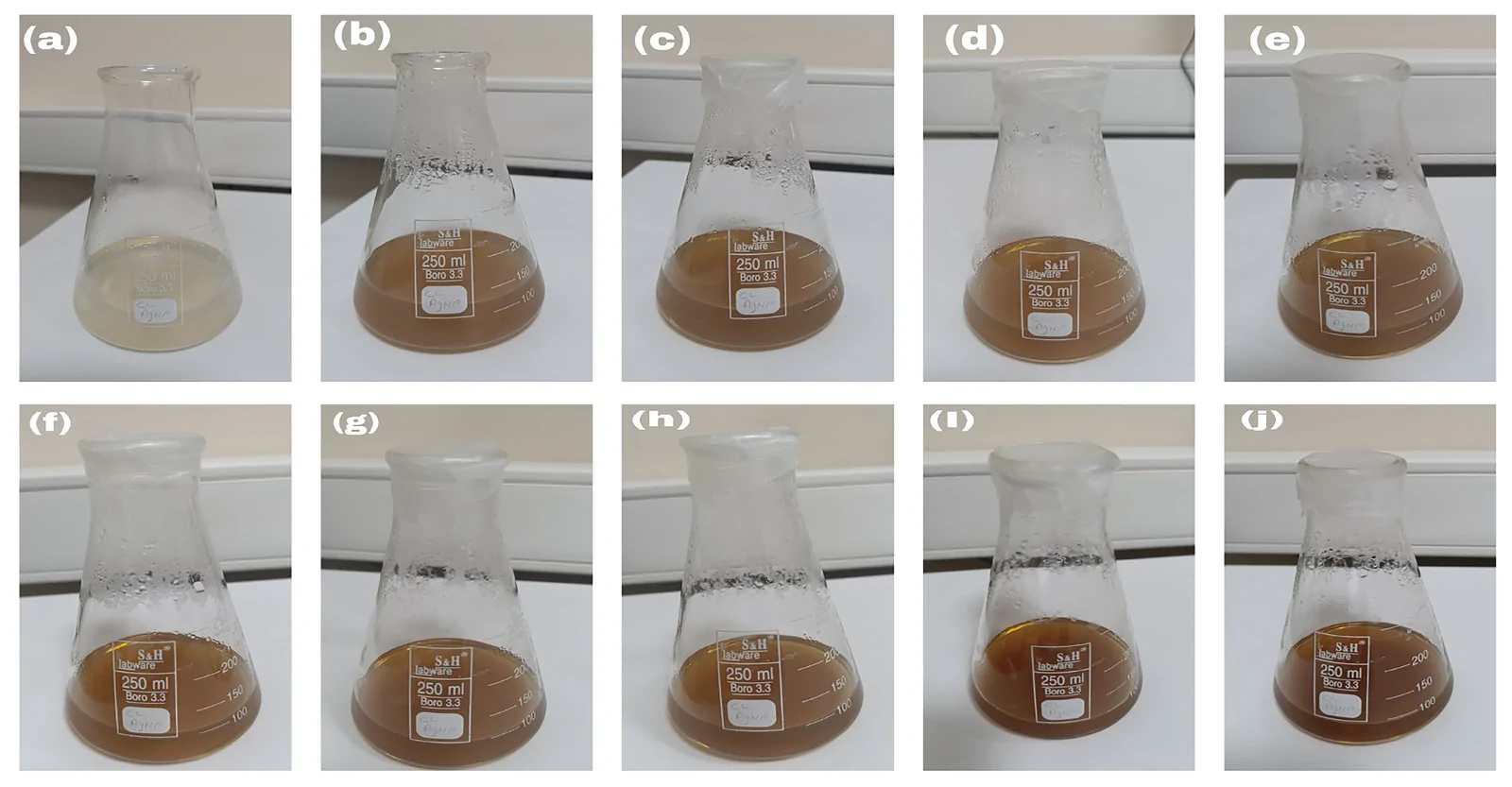
Color changes of silver nanoparticles synthesized using *Chaetomorpha linum* extract depending on reaction time: (**a**) the initial stage of the reaction, (**b**) the first color change, (**c**) after 1 h, (**d**) after 2 h, (**e**) after 4 h, (**f**) after 6 h, (**g**) after 8 h, (**h**) after 12 h, (**i**) after 24 h, and (**j**) the change in the color intensity of *Chaetomorpha linum* extract after 48 h.

**Figure 3:**
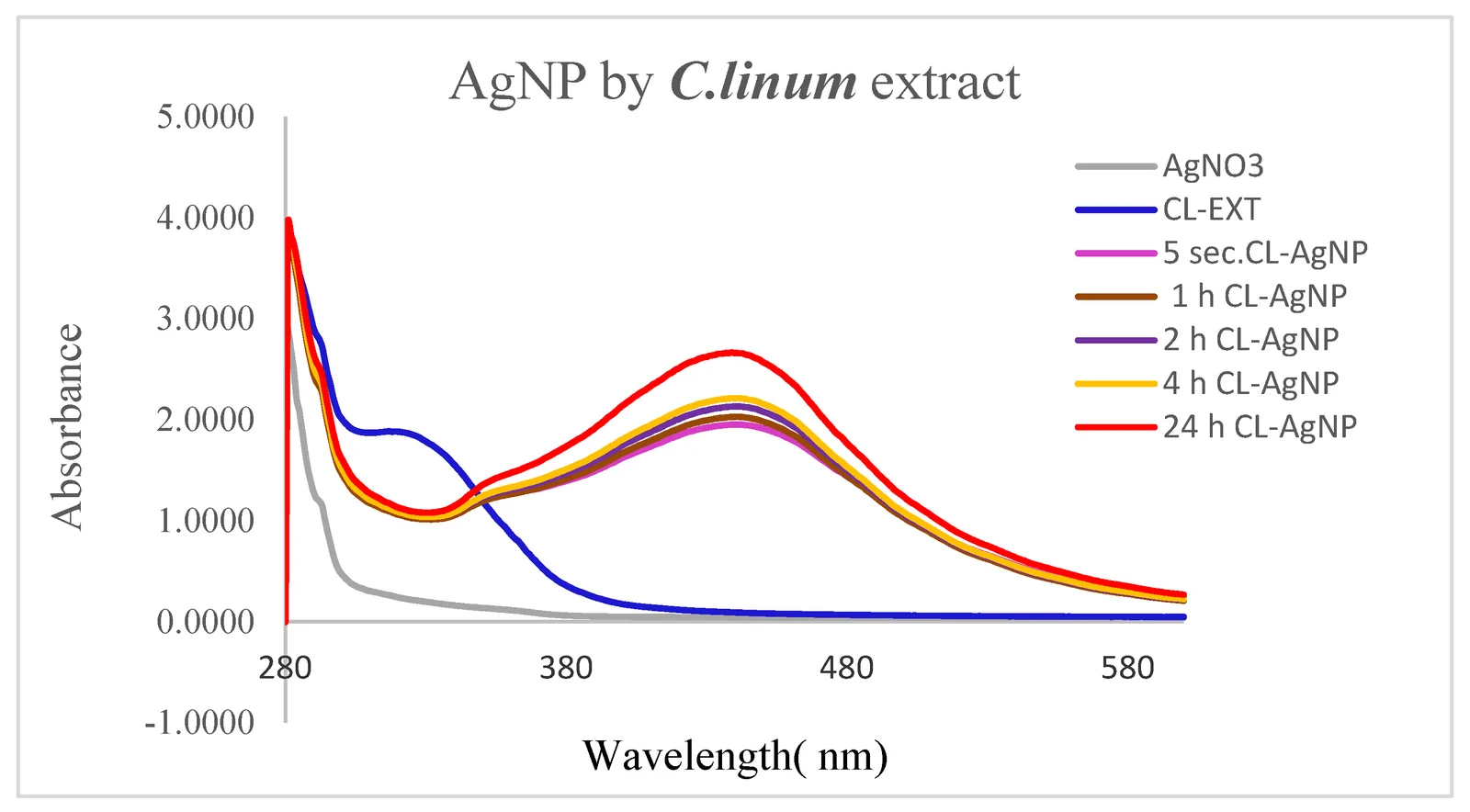
UV–Vis absorbance spectra illustrating the time-dependent formation of silver nanoparticles synthesized using *Chaetomorpha linum* extract.

**Figure 4:**
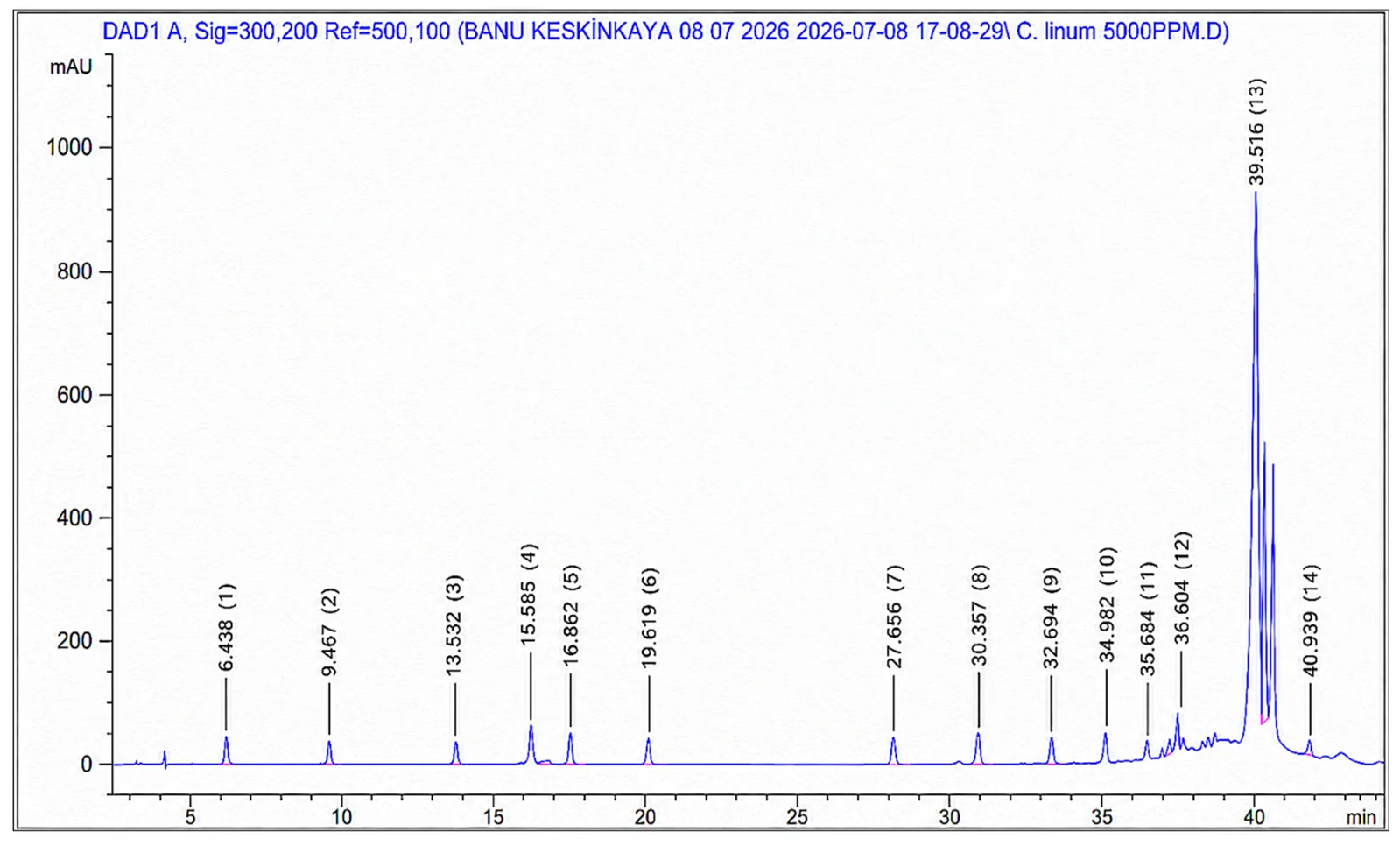
A representative HPLC-DAD chromatogram of the aqueous extract of *Chaetomorpha linum* showing the identified phenolic compounds.

**Figure 5:**
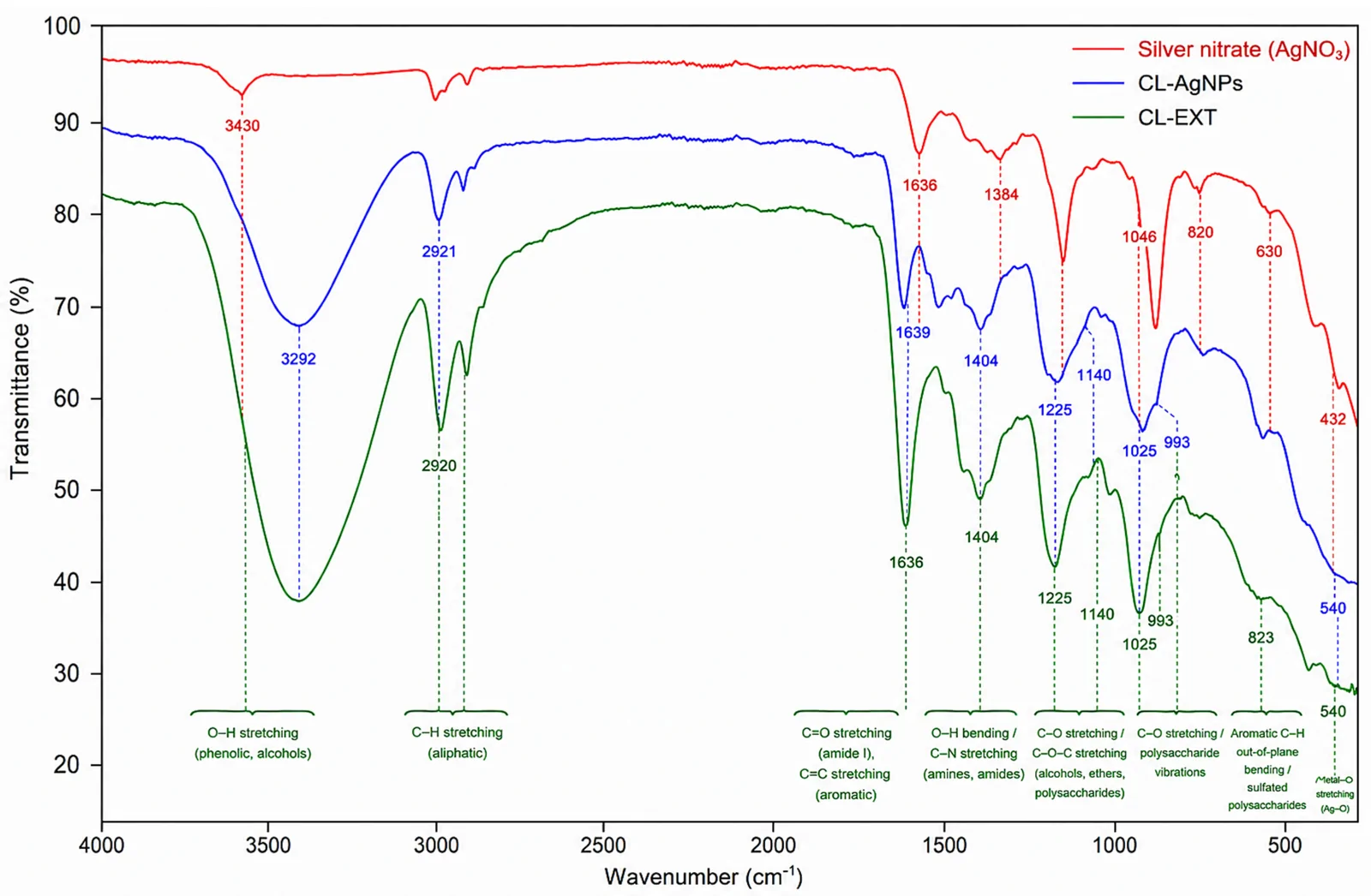
FTIR spectrum of silver nanoparticles biosynthesized using *Chaetomorpha linum* extract, illustrating characteristic functional groups responsible for bioreduction and capping.

**Figure 6:**
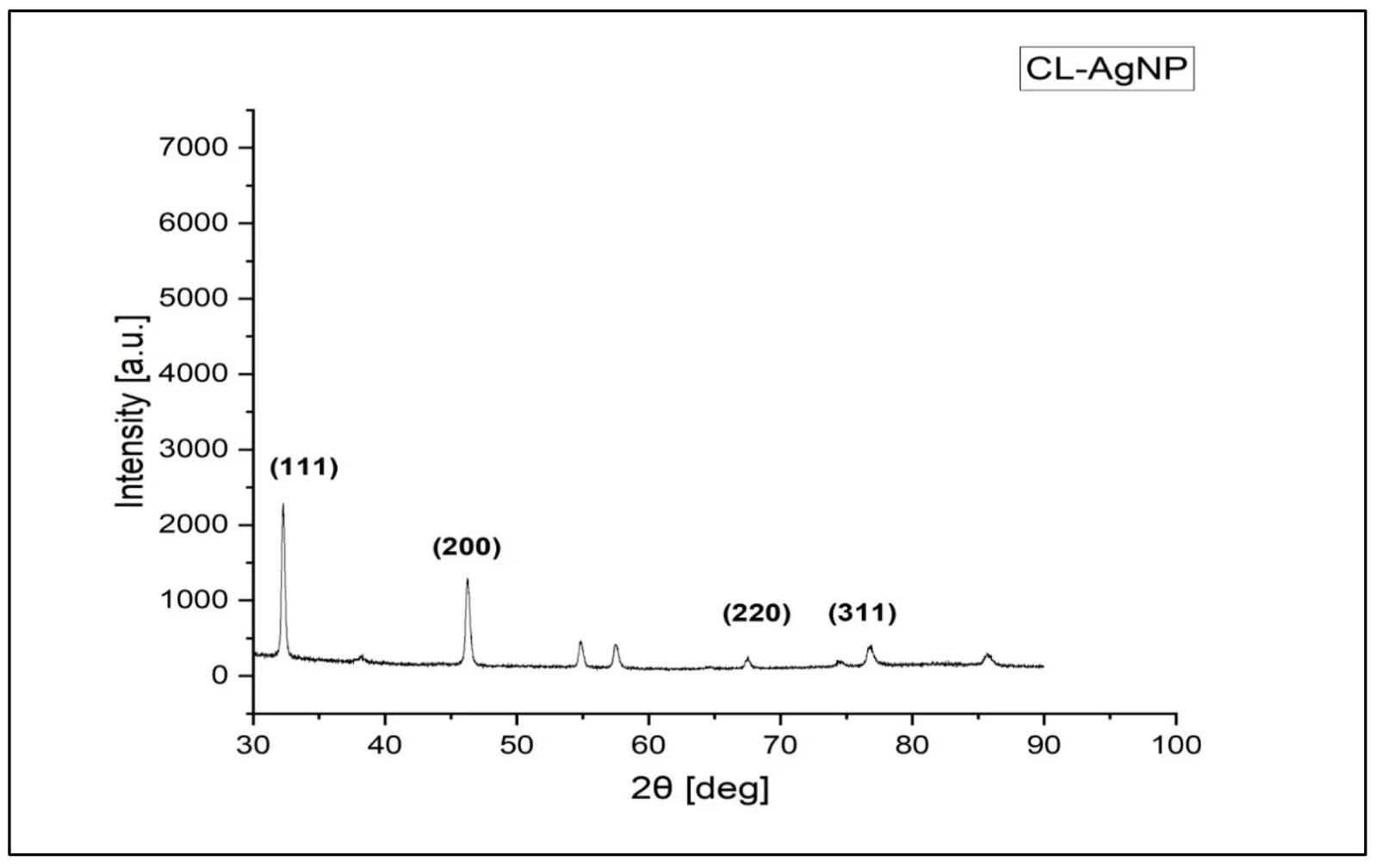
The X-ray diffraction (XRD) pattern of biosynthesized CL-AgNPs. The indexed diffraction peaks correspond to the (111), (200), (220), and (311) crystallographic planes of face-centered cubic (fcc) silver and are consistent with JCPDS Card No. 04-0783.

**Figure 7:**
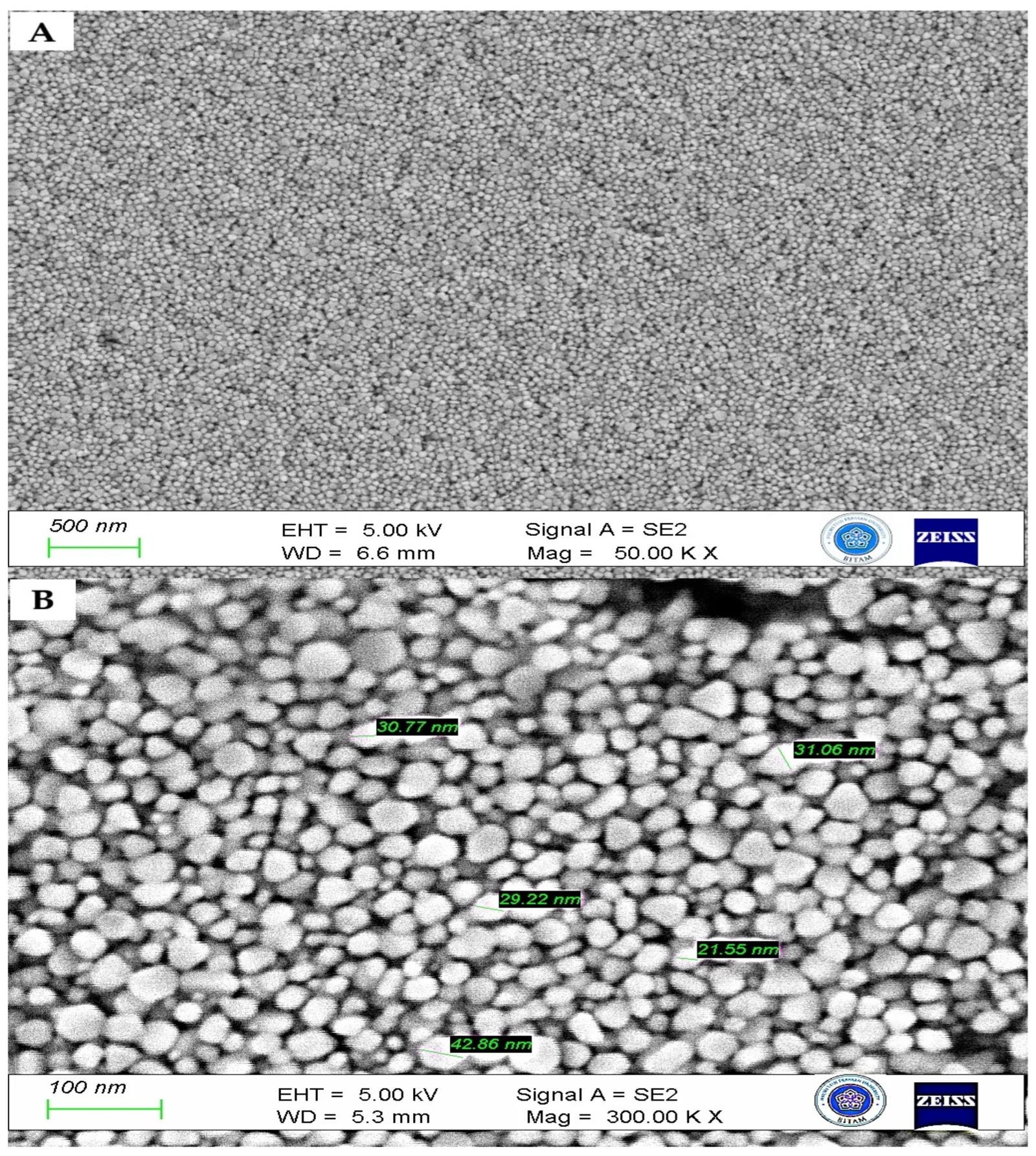
FESEM micrographs of biosynthesized CL-AgNPs recorded at two different magnifications: (**A**) 50,000× and (**B**) 300,000×. The higher-magnification image clearly demonstrates the predominantly spherical to quasi-spherical morphology of the nanoparticles and includes representative particle size measurements obtained using ImageJ software. Particle size analysis was performed on 50 well-dispersed primary nanoparticles, yielding an average particle diameter of 31.09 ± 2.52 nm (mean ± SD, *n* = 50) with a size range of 21.26–42.90 nm.

**Figure 8:**
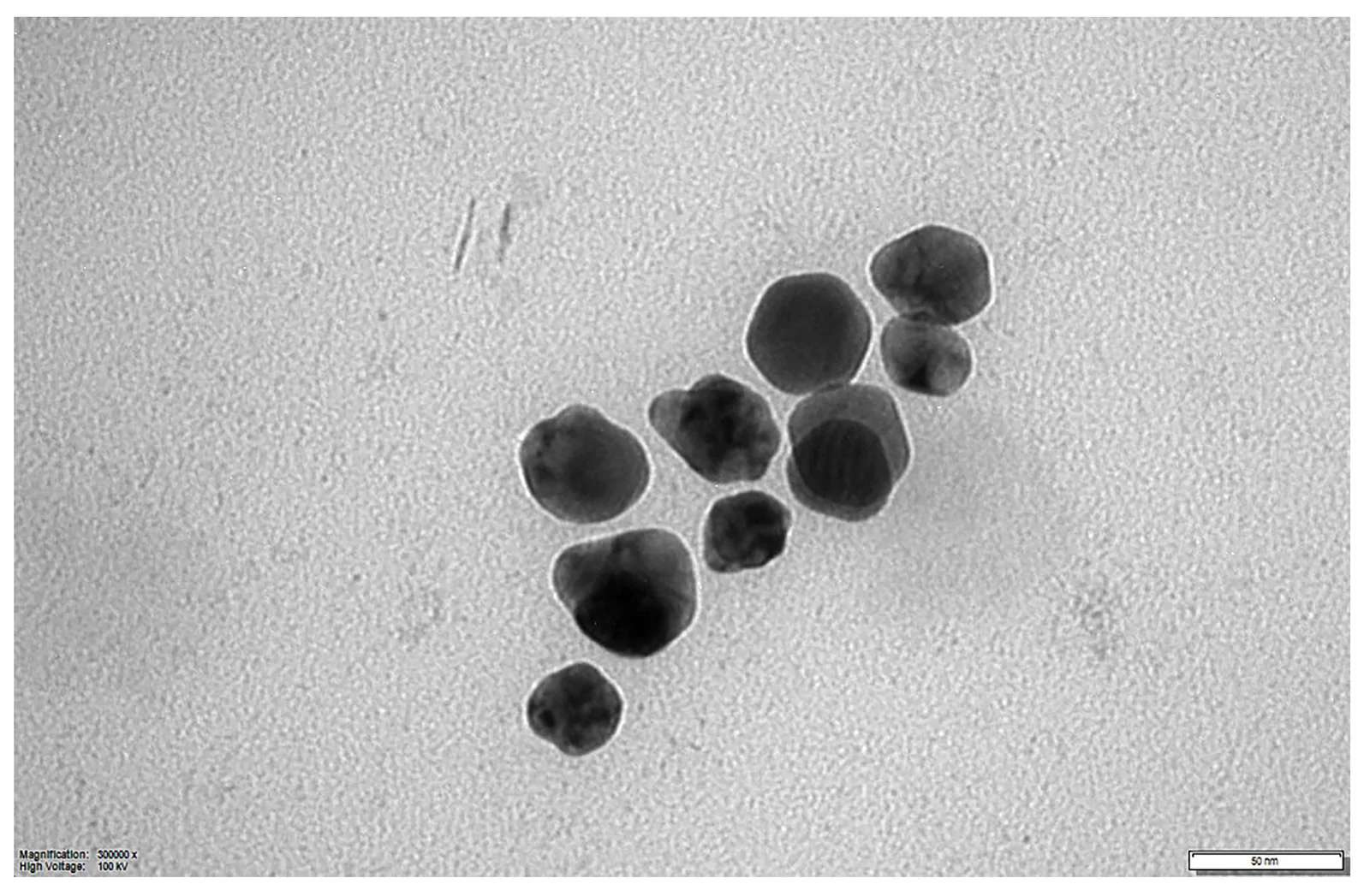
A TEM micrograph of biosynthesized CL-AgNPs showing predominantly discrete primary nanoparticles. Particle size analysis performed using ImageJ (*n* = 8) yielded an average diameter of 30.87 ± 2.94 nm, corroborating the FESEM measurements.

**Figure 9:**
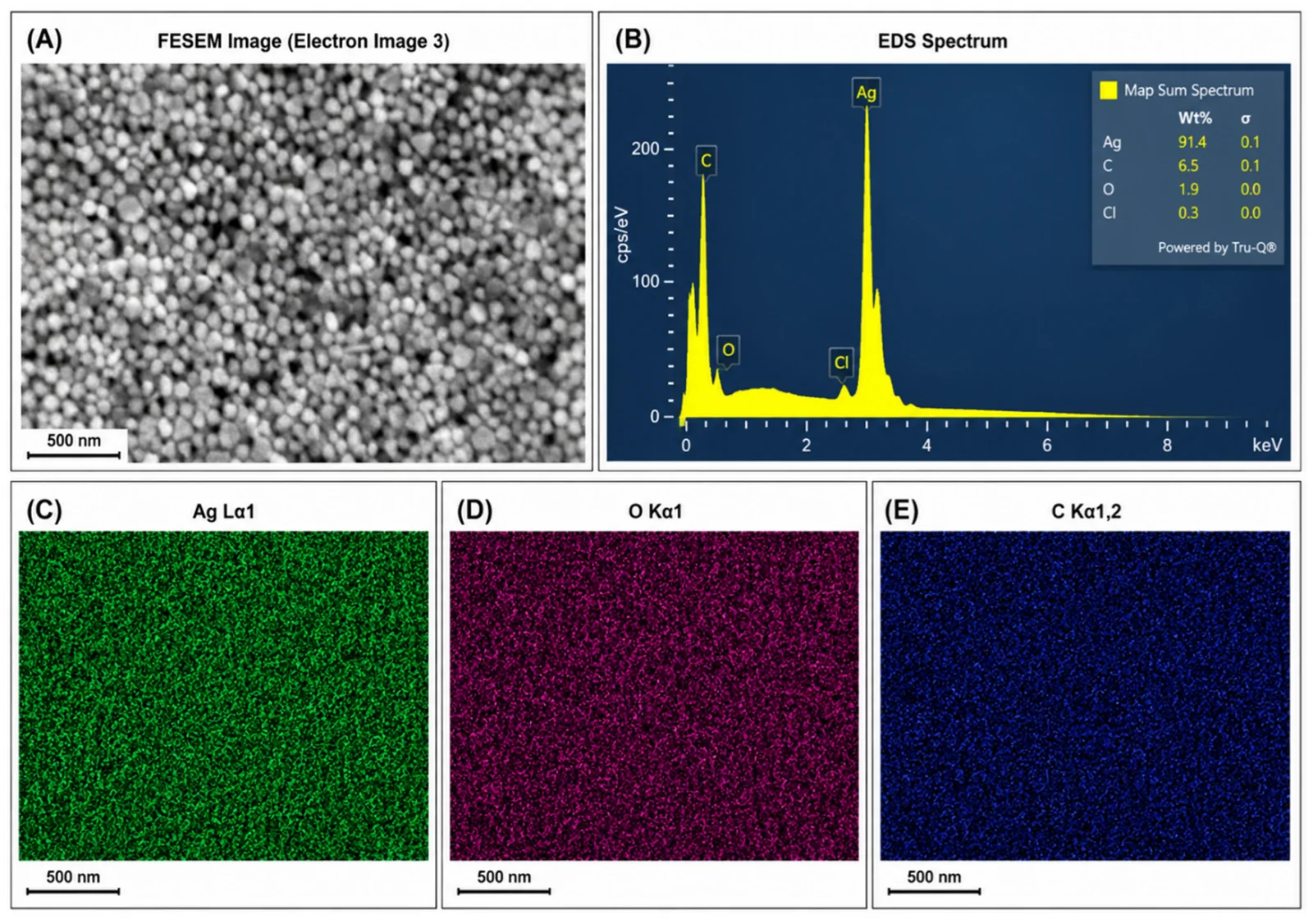
A FESEM micrograph (**A**), EDS spectrum (**B**), and corresponding elemental distribution maps of Ag (**C**), O (**D**), and C (**E**) for the biosynthesized AgNPs. The elemental maps provide qualitative information on the spatial distribution of the detected elements and complement the elemental composition determined by EDS analysis.

**Figure 10:**
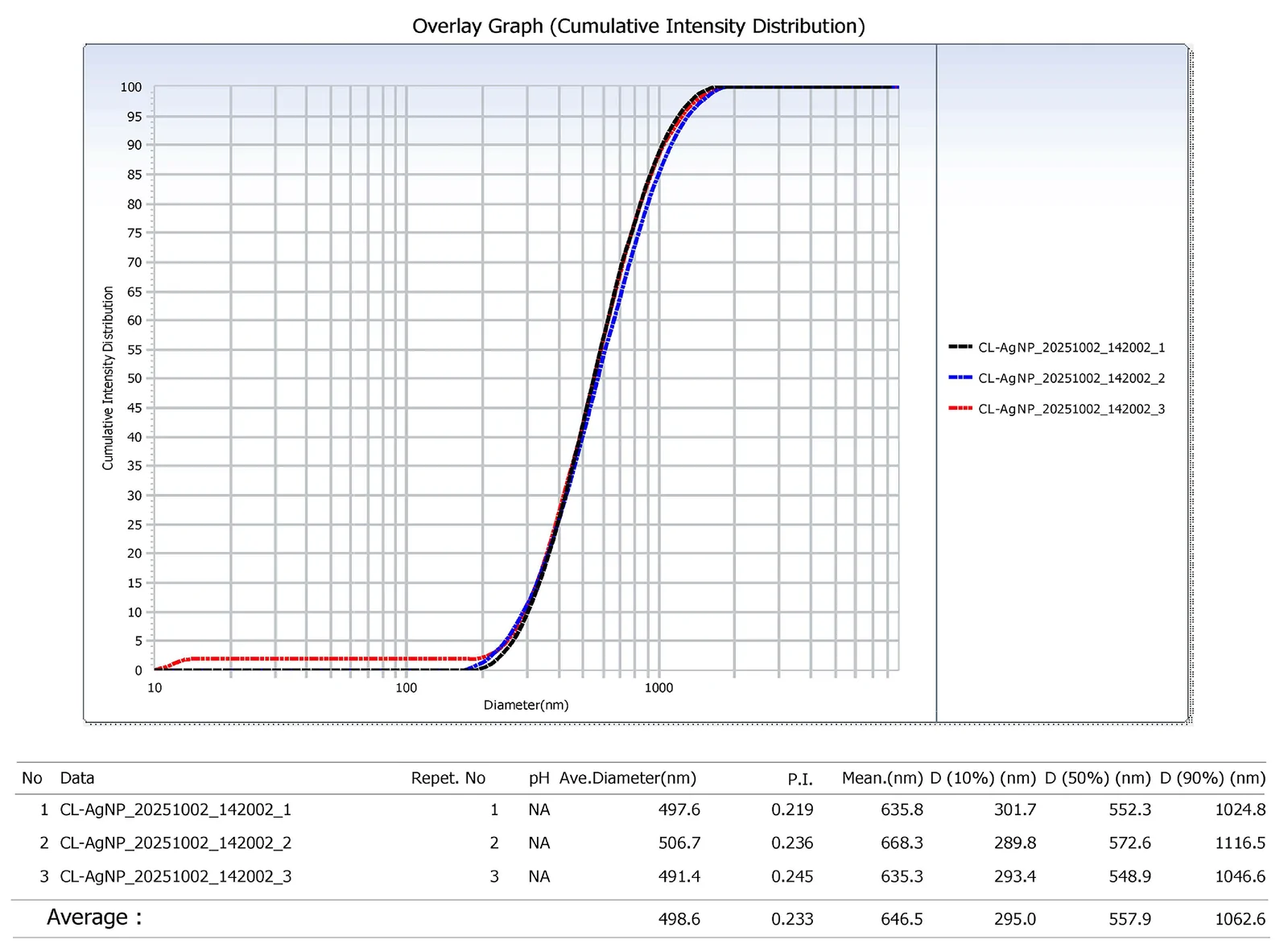
Cumulative intensity distribution curves and particle size distribution parameters of biosynthesized *Chaetomorpha linum*-mediated silver nanoparticles (CL-AgNPs) determined by dynamic light scattering (DLS). Three independent measurements exhibited comparable particle size distributions, with a mean hydrodynamic diameter of 646.5 ± 18.9 nm and particle size distribution percentiles of D10 = 295.0 ± 6.1 nm, D50 = 557.9 ± 12.8 nm, and D90 = 1062.6 ± 47.9 nm (mean ± SD, *n* = 3).

**Figure 11:**
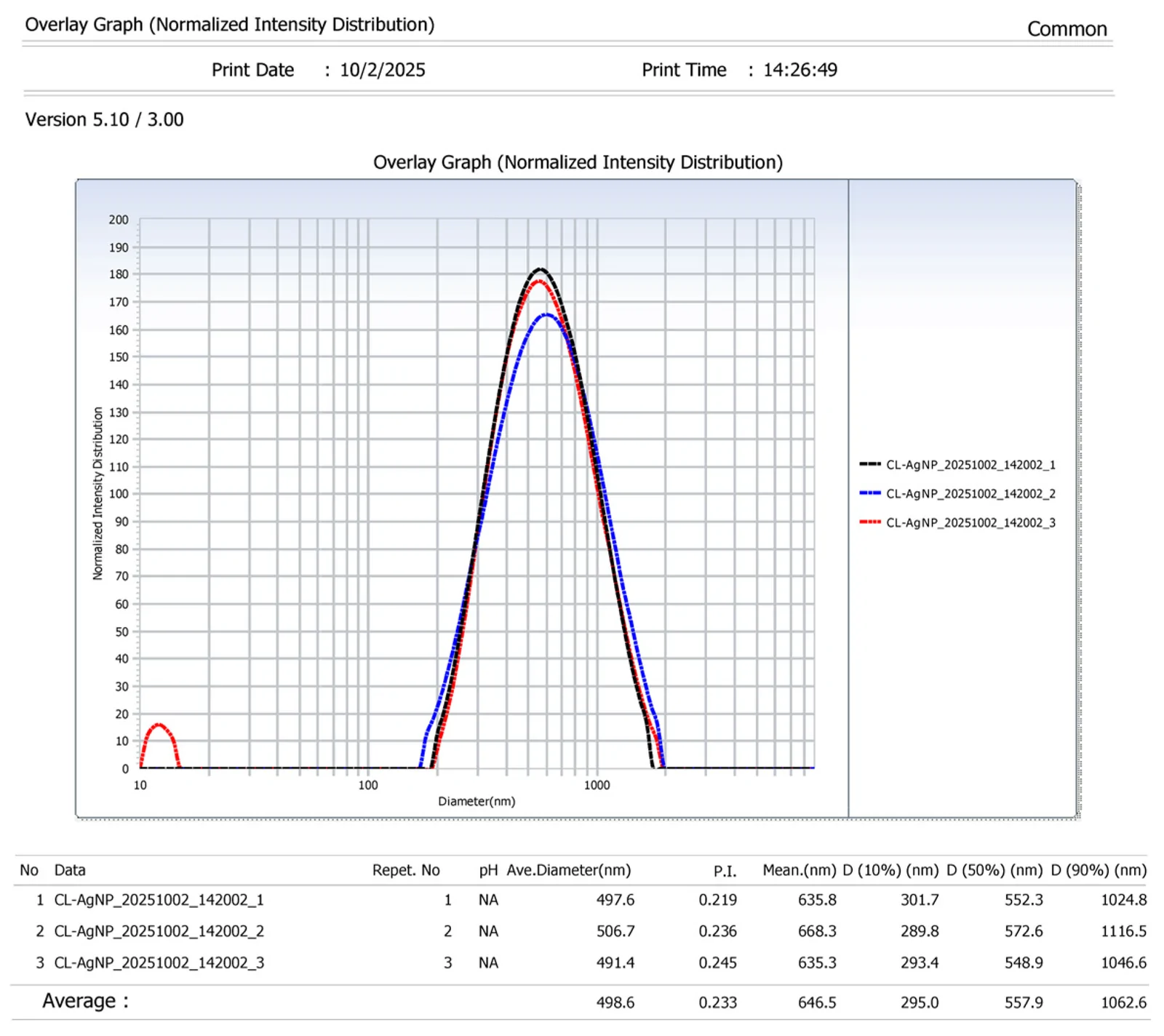
Instrumental DLS particle size distribution by normalized intensity of biosynthesized AgNPs obtained from three independent measurements.

**Figure 12:**
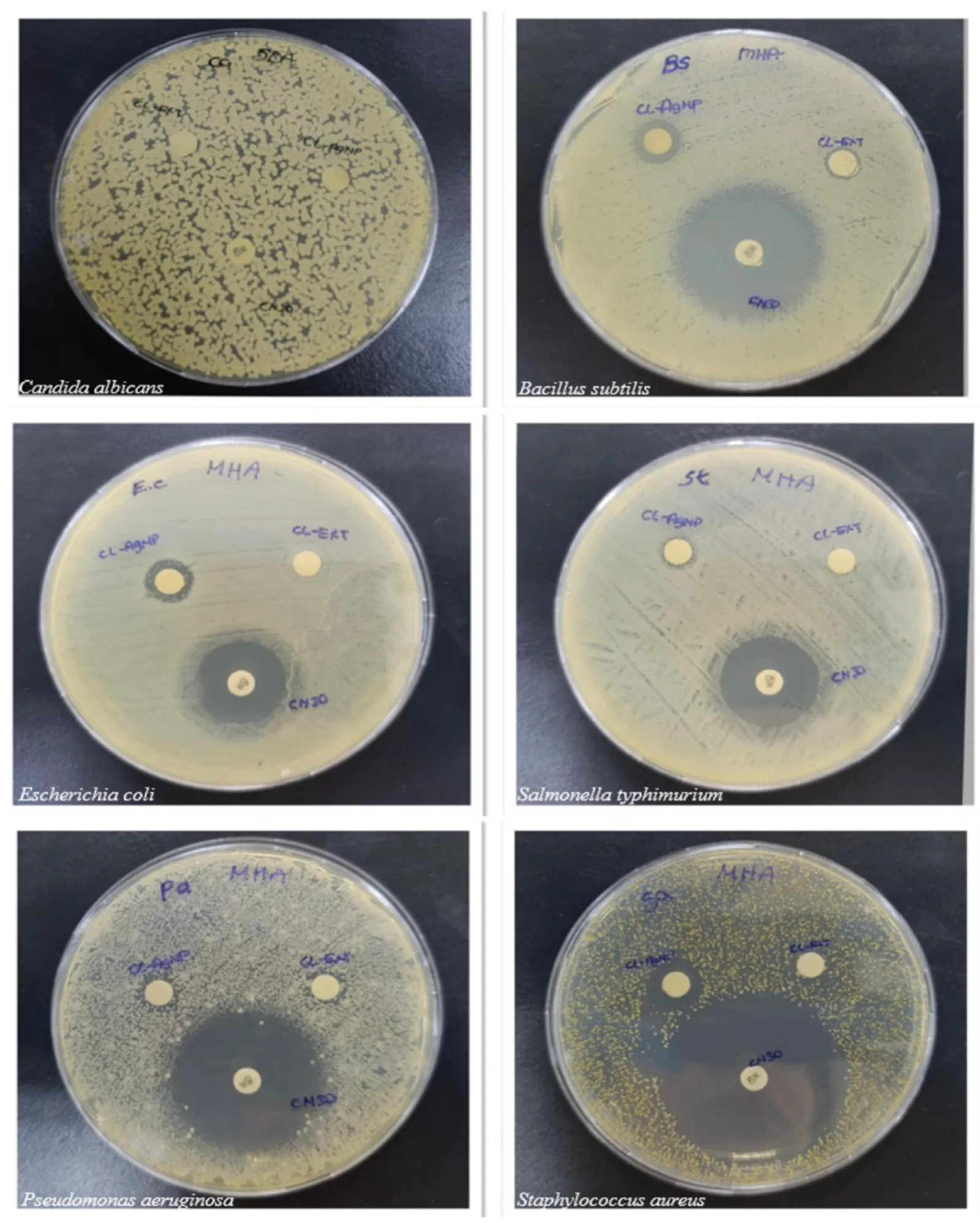
Representative inhibition zones obtained via the disk diffusion method for CL-EXT, CL-AgNPs and gentamicin (GND) against selected bacterial and fungal strains. CL-AgNPs exhibited greater antimicrobial activity than CL-EXT, particularly against *Staphylococcus aureus* and *Bacillus subtilis*.

**Table 1:** Systematic taxonomy of the genus *Chaetomorpha linum* (O.F. Müller) Kützing 1845 (adapted from AlgaeBase) [3].

Rank	Classification
Domain	Eukaryota
Kingdom	Plantae
Subkingdom	Viridiplantae
Infrakingdom	Chlorophyta
Phylum	Chlorophyta
Subphylum	Chlorophytina
Class	Ulvophyceae
Order	Cladophorales
Family	Cladophoraceae
Genus	*Chaetomorpha*
Species	*Chaetomorpha linum* (O.F. Müller) Kützing

**Table 2:** Phenolic compounds identified in the aqueous extract of *Chaetomorpha linum* by HPLC-DAD analysis.

Compound	Retention Time (min)	Concentration (ng/µL)
Catechin hydrate	6.438	0.267
Caffeic acid	9.467	0.645
4-Hydroxybenzoic acid	13.532	3.576
Vanillin	15.585	4.661
*p*-Coumaric acid	16.862	0.064
Rutin	19.619	10.062
Naringin	27.656	6.172
Rosmarinic acid	30.357	0.142
Resveratrol	32.694	0.075
Quercetin	34.982	1.046
*trans*-Cinnamic acid	35.684	1.115
Naringenin	36.604	4.441
Chrysin	39.516	821.655
Flavones	40.939	5.064

Concentrations of the identified phenolic compounds are expressed as ng/µL. Only detected compounds are included.

**Table 3:** Inhibition zone diameters (mm) of CL-EXT, biosynthesized silver nanoparticles (CL-AgNPs), and positive controls against the tested bacterial and fungal strains determined via the disk diffusion method.

Test Microorganism	CL-EXT	CL-AgNP	Positive Control
*S. aureus*	- *	13 mm	42 mm
*B.subtilis*	0.8 mm	10 mm	32 mm
*E.coli*	-	-	21 mm
*P. aeruginosa*	-	-	30 mm
*S. typhimurium*	-	0.7 mm	22 mm
*C. albicans*	-	-	-

* -: Not detected. Gentamicin was used as the positive control for bacterial strains, whereas fluconazole was used as the positive control for *Candida albicans*.

**Table 4:** Minimum inhibitory concentration (MIC, μg/mL) values of CL-EXT, CL-AgNPs, and the positive controls against the tested microorganisms.

Test Microorganism	CL-EXT	CL-AgNP	Positive Control
*S. aureus*	- *	1.56	0.39
*B.subtilis*	-	3.125	0.19
*E.coli*	6.25	1.56	0.19
*P. aeruginosa*	-	3.125	0.78
*S. typhimurium*	-	3.125	1.56
*C. albicans*	-	1.56	1.56

* -: Not detected. Gentamicin was used as the positive control for bacterial strains, whereas fluconazole was used as the positive control for *Candida albicans*.

**Table 5:** Antioxidant activity profiles of CL-EXT and CL-AgNPs determined by DPPH, ABTS, CUPRAC and metal chelating assays.

		Antioxidant Activity							
		DPPH^•^ Assay		ABTS^•+^ Assay		CUPRAC Assay		Metal Chelating Assay	
		Inhibition (%) ^a^	IC_50_ (µg/mL) ^b^	Inhibition (%) ^a^	IC_50_ (µg/mL) ^b^	Absorbance ^c^	A_0.50_ (µg/mL) ^d^	Inhibition (%) ^a^	IC_50_ (µg /mL) ^b^
Samples	CL-EXT	56.34 ± 0.21	32.40 ± 0.18	65.77± 0.26	29.80 ± 0.24	3.18 ± 0.04	35.60 ± 0.22	286.88± 0.48	45.30 ± 0.35
	CL-AgNPs	78.65 ±0.98	24.67± 0.43	80.12 ± 0.13	17.85± 0.62	4.74± 0.02	28.87± 0.91	362.35 ± 0.24	31.93± 0.47
Standards	BHT	90.12 ± 0.16	18.10 ± 0.30	86.20 ± 0.28	11.40 ± 0.42	3.55 ± 0.04	21.90 ± 0.22	-	-
	BHA	92.35 ± 0.14	17.60 ± 0.25	88.15 ± 0.30	10.90 ± 0.38	3.72 ± 0.03	20.80 ± 0.20	-	-
	EDTA							95.80 ± 0.35	2.90 ± 0.05

^a^: Inhibition values % of 400 µg/mL concentration of the extracts are given as a mean ±SD of three parallel measurements. ^b^: IC_50_ values are given as a mean ±SD of three parallel measurements. ^c^: Absorbance values of 400 µg/mL concentration of the extracts are given as a mean ±SD of three parallel measurements. ^d^: A_0.50_ values are given as a mean ±SD of three parallel measurements.

## Data Availability

The original contributions presented in this study are included in the article. Further inquiries can be directed to the corresponding author.

## Supplementary Materials

The following supporting information can be downloaded at: https://www.mdpi.com/article/10.3390/ma19194119/s1. Figure S1. Propsed mechanism of silver nanoparticle biosynthesis mediated by *Chaetomorpha linum* aqueous extract.
